# Optimizing reaction conditions for the light-driven hydrogen evolution in a loop photoreactor

**DOI:** 10.3762/bjoc.20.9

**Published:** 2024-01-16

**Authors:** Pengcheng Li, Daniel Kowalczyk, Johannes Liessem, Mohamed M Elnagar, Dariusz Mitoraj, Radim Beranek, Dirk Ziegenbalg

**Affiliations:** 1 Institute of Chemical Engineering, Ulm University, Albert-Einstein-Allee 11, 89081 Ulm, Germany; 2 Institute of Electrochemistry, Ulm University, Albert-Einstein-Allee 47, 89081 Ulm, Germany

**Keywords:** loop photoreactor, parametric study, photocatalytic hydrogen evolution, polymeric carbon nitride, solar energy storage

## Abstract

Photocatalytic hydrogen production from water is a promising way to fulfill energy demands and attain carbon emission reduction goals effectively. In this study, a loop photoreactor with a total volume of around 500 mL is presented for the photocatalytic hydrogen evolution using a Pt-loaded polymeric carbon nitride photocatalyst under 365 nm irradiation in the presence of sacrificial reducing agents. The fluid flow pattern of the developed photoreactor was characterized experimentally and the photon flux incident to the loop photoreactor was measured by chemical actinometry. The system displayed exceptional stability, with operation sustained over 70 hours. A design of experiment (DOE) analysis was used to systematically investigate the influence of key parameters – photon flux, photocatalyst loading, stirring speed, and inert gas flow rate – on the hydrogen generation rate. Linear relationships were found between hydrogen evolution rate and photon flux as well as inert gas flow rate. Photocatalyst loading and stirring speed also showed linear correlations, but could not be correctly described by DOE analysis. Instead, linear single parameter correlations could be applied. Notably, the loop photoreactor demonstrated an external photon efficiency up to 17 times higher than reported in literature studies, while scaling the reactor size by a factor of 10.

## Introduction

The world is in the midst of the first truly global energy crisis with unprecedented breadth and complexity [1]. Meanwhile, the rise of the world’s population and economic growth demands more energy. Of all energy sources, fossil fuels have been dominating over 80% of the global energy market for decades [1–3]. However, the combustion of fossil fuels contributes greatly to environmental degradation, global warming, and air pollution, which further damages public health [4–6]. Given the nature of how fossil fuels are formed, which takes millions of years to come to be [7], they are considered limited resources and nonrenewable compared to the current consumption rate [8]. Therefore, a pressing need exists to transition towards renewable energy sources to ensure sustainable energy development and environmental preservation. Solar energy stands out as the most compelling renewable energy source, holding the potential to fully satisfy the energy requirements of humanity [9]. The reliance on geographic locations and seasonal changes, however, makes it difficult to maintain a constant supply of solar energy year-round [10]. To harness solar energy in a proper way, solar energy can be converted to hydrogen fuels. Hydrogen as an energy carrier has zero carbon emission and a high energy density [11–12]. While significant efforts are directed towards developing effective photocatalysts for solar water splitting [13–16], a crucial emphasis must also be placed on upscaling reactors and catalytic systems to facilitate the transition to cleaner energy sources.

There are mainly three categories of solar to hydrogen generation methods based on semiconductors, namely photovoltaic-powered electrolysis (PV-E), photoelectrochemical (PEC) processes, and photocatalysis. Upon upscaling, both PV-E and PEC reactors exhibit pH gradients at electrodes and elevated solution electrical resistivity. These challenges arise from the substantial separation between reduction and oxidation sites, alongside mass transport restrictions in the liquid phase due to extended ion transport distances. These effects cause significant potential losses, eventually reducing the efficiency of the reactors. The addition of large amounts of supporting electrolyte and vigorous circulation of the electrolyte solution also complicates the scale-up process [17–18]. On the contrary, expanding photocatalytic systems, particularly those employing powdered photocatalysts, to a larger scale is notably more straightforward [17–18]. However, complications arise when attempting to scale up reactors solely based on geometric similarity, as alterations in mixing and hydrodynamic attributes occur during the process of scale-up [19–20]. Therefore, a suitable reactor, which ensures excellent fluid dynamics and transport properties during scale-up, is needed for photocatalytic hydrogen generation.

Loop reactors utilize partial inner circulation of the chosen reaction medium around an internal concentrically fitted draft tube, and are widely used in industry, such as microbiology fermentation and Fischer–Tropsch synthesis [21–22]. This type of reactors provides very high mass transfer rates, well-defined fluid flow patterns, and a simple design [23–24]. The construction and operation of loop reactors are simple. Furthermore, investment and operational costs are typically low. For these reasons, the suitability of a loop reactor was studied in this work for the photocatalytic hydrogen generation process as a potentially scalable photoreactor concept.

Suitable light sources are one of the requirements for photocatalytic studies, as they directly influence the performance of photocatalytic reactions. While the ultimate goal is to utilize sunlight directly for photocatalytic hydrogen generation, the limited intensity of sunlight poses challenges [25]. Beside this, identifying photocatalysts capable of efficient performance across broad sunlight wavelength ranges proves to be a complex endeavor [26–28]. Therefore, artificial light sources are typically applied for lab testing of photocatalytic reactions. Compared to conventional light sources, light-emitting diodes (LEDs) have plenty of advantages including long life span (up to 100 000 h), robustness, compact size, and high energy efficiency, which make LEDs very attractive as a light source for photocatalytic hydrogen generation studies [29].

**Scheme 1:**
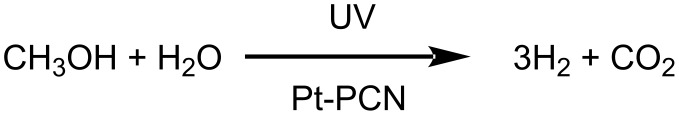
Photocatalytic hydrogen evolution with Pt-loaded polymeric carbon nitride.

In this work, a loop photoreactor for scaling up the photocatalytic hydrogen generation is designed and equipped with 365 nm LEDs. Fluid dynamics in the loop photoreactor are characterized using a color tracer mixing experiment and image analysis. Photonic characterization is performed with the ferrioxalate actinometer. Finally, the photocatalytic hydrogen evolution with Pt-loaded polymeric carbon nitride (Pt-PCN) powder using methanol as sacrificial agent (Scheme 1) is studied as a benchmark in the designed loop photoreactor. Four parameters, namely photon flux, photocatalyst loading, stirring speed, and inert gas flow rate, are investigated for their effect on the activity of the hydrogen evolution. The efficiency of the loop photoreactor is also compared with results from literature obtained in a Schlenk tube system [30].

## Results and Discussion

### Photocatalyst characterization

**Figure 1:**
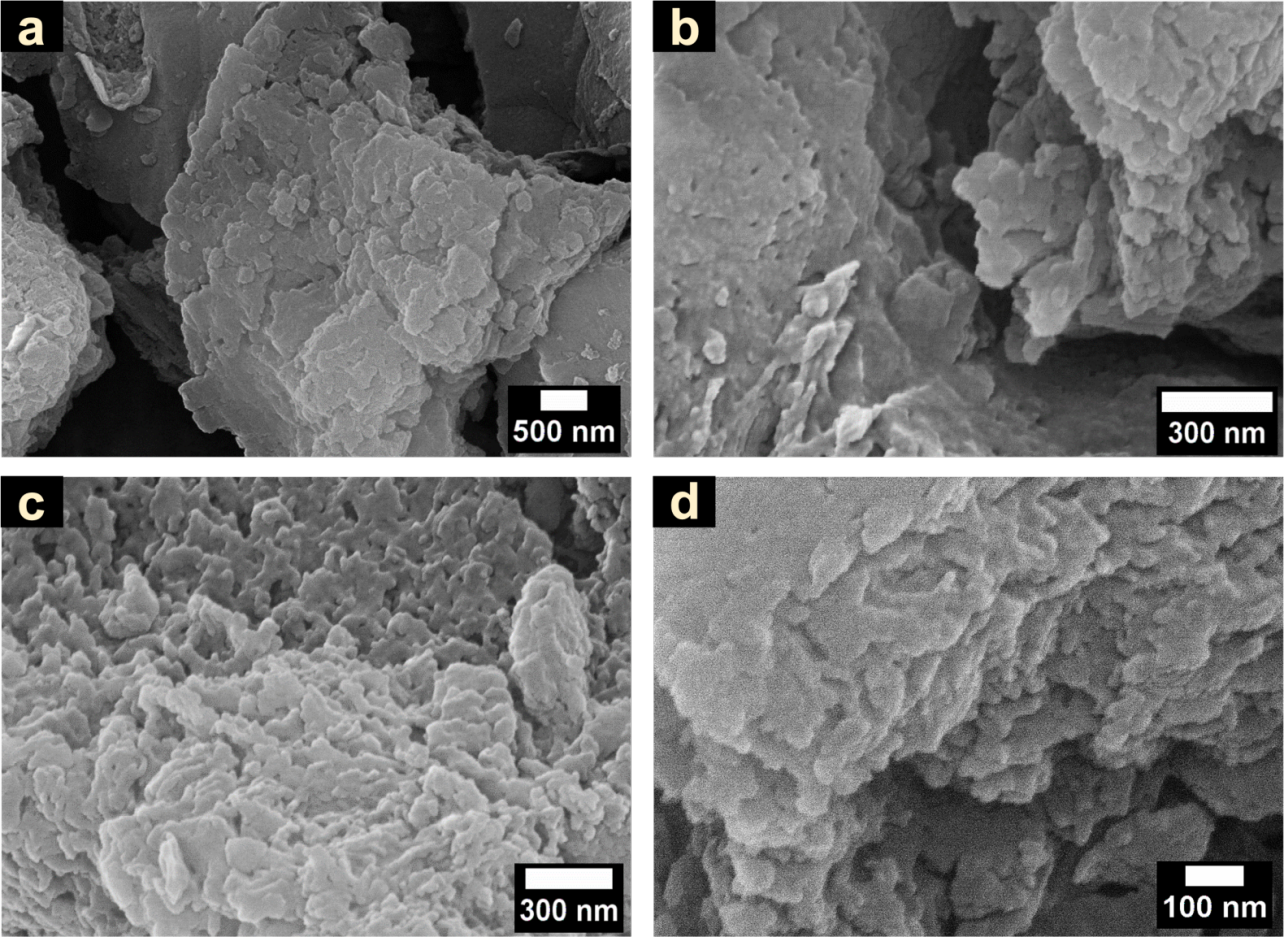
SEM images of the Pt-PCN photocatalyst at different magnifications.

**Figure 2:**
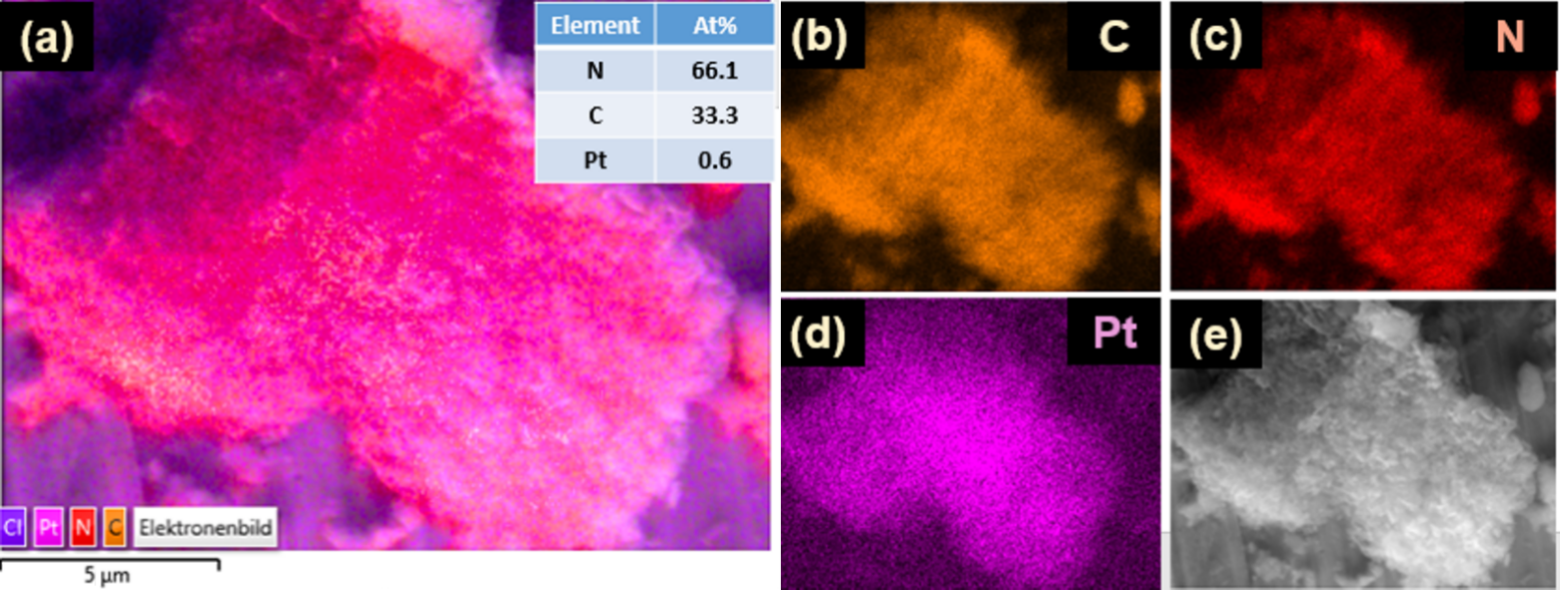
SEM-EDX elemental mapping images of C, N, and Pt on a particle.

The surface morphology of the Pt-PCN photocatalyst has been analyzed by scanning electron microscopy (SEM) at different magnifications, as shown in Figure 1a–d. The images reveal a layered structure exhibiting a rough surface with agglomerated, irregular, and dense particles. Figure 2a–e shows the elemental mapping analysis of Pt-PCN conducted using SEM-EDX. A homogeneous distribution of Pt (Figure 2d) was found across the surfaces of the Pt-PCN, confirming their uniformity. To ensure that the homogeneous distribution of Pt on the surface of PCN is not random, multiple particles were analyzed using SEM-EDX (see Supporting Information File 1, Figure S1), which shows the uniform distribution of Pt on the surfaces of PCN over all particles. Moreover, the EDX elemental composition analysis indicates that the Pt content is 0.6 atom %.

### Reactor characterization

#### Fluid flow pattern

**Figure 3:**
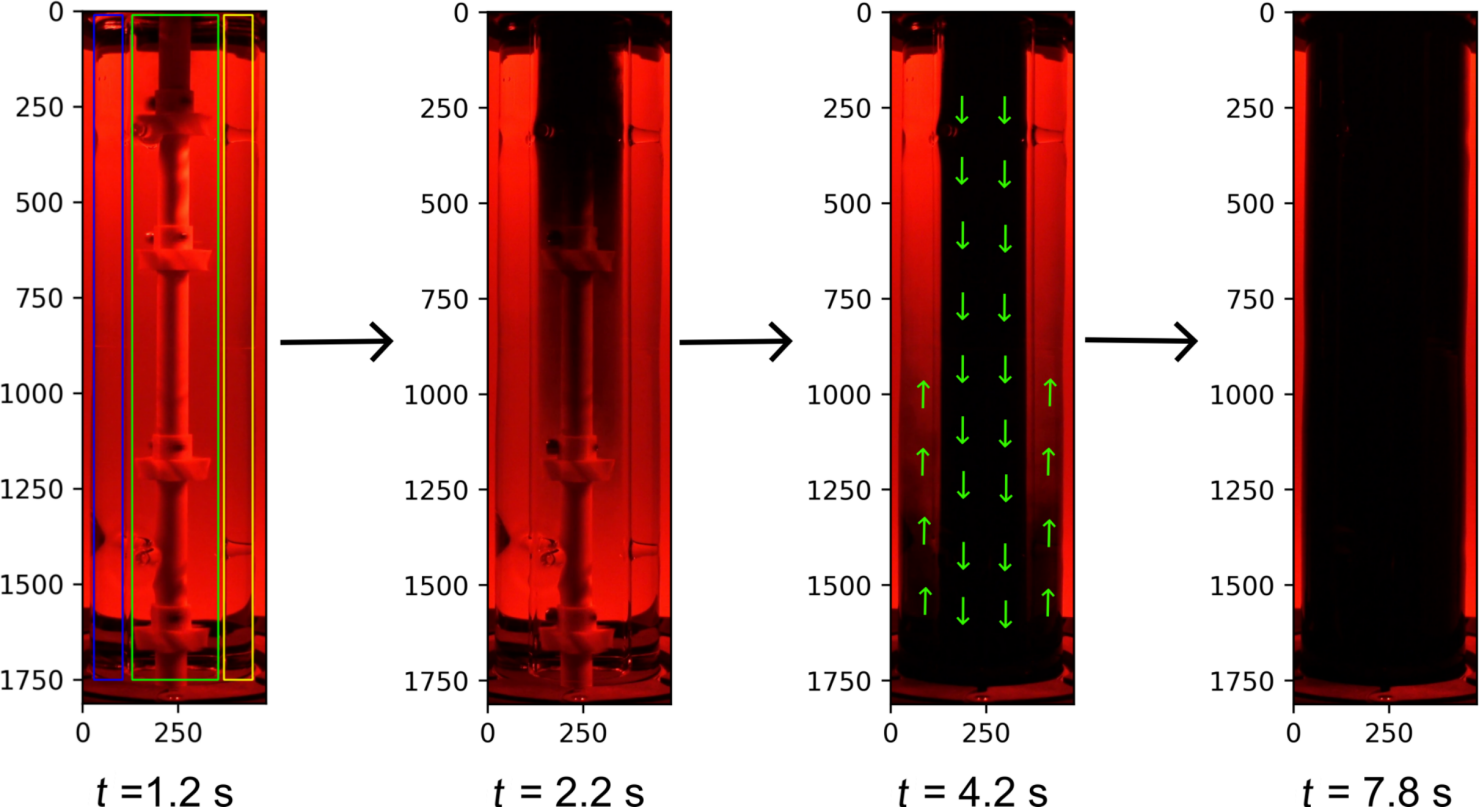
Exemplary results of the mixing experiments using methylene blue to visualize and quantify fluid flow pattern in the loop photoreactor at stirring speed of 560 rpm.

Figure 3 shows four representative frames of a mixing experiment at times of 1.2, 2.2, 4.2, and 7.8 s using a stirring speed of 560 rpm. The regions of interests (ROIs) shown on frame of 1.2 s are denoted as left side (blue ROI), draft tube (green ROI), and right side (yellow ROI). The color of water changed first in the draft tube and then outside the draft tube until the mixing was complete. Thus, the liquid was first sucked into the draft tube and flowed through the draft tube downwards until it reached the bottom of the reactor, where it was redirected to flow upward outside the draft tube back to the top of the reactor. The arrows on the frame of 4.2 s show the fluid flow direction. This confirms the desired operation of the reactor design concept of a guided inner circulation around the draft tube.

**Figure 4:**
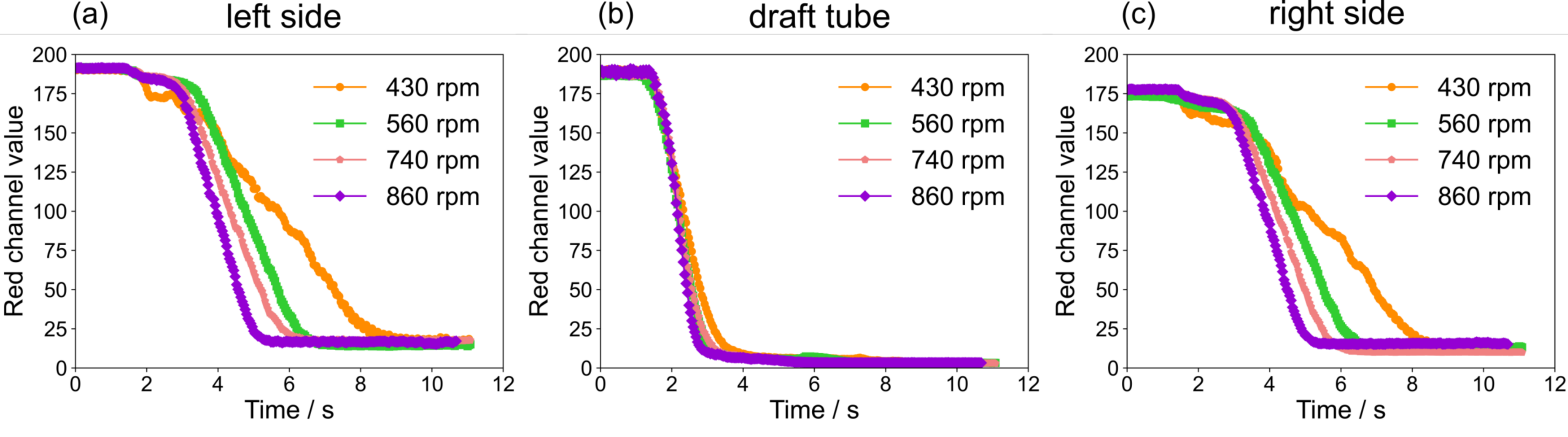
Red channel values of ROIs with different stirring speeds: (a) left space between reactor and draft tube, (b) in draft tube, (c) right space between reactor and draft tube.

Figure 4 shows the red channel values of ROIs for the mixing experiments with different stirring speeds (430, 560, 740, and 860 rpm). The mixing time in the draft tube only slightly depends on the stirring speed. However, the mixing time outside of the draft tube shows a clear dependence on the stirring speed. As expected, both sides of the reactor show an almost identical mixing behavior. Data for a stirring speed of 430 rpm shows pronounced fluctuation. This is because not the whole methylene blue solution is immediately sucked into the draft tube. Instead, parts of the tracer solution are swirling above the draft tube, eventually being sucked slowly into the draft tube and causing the observed fluctuations (see Supporting Information File 2, loop_430rpm).

**Figure 5:**
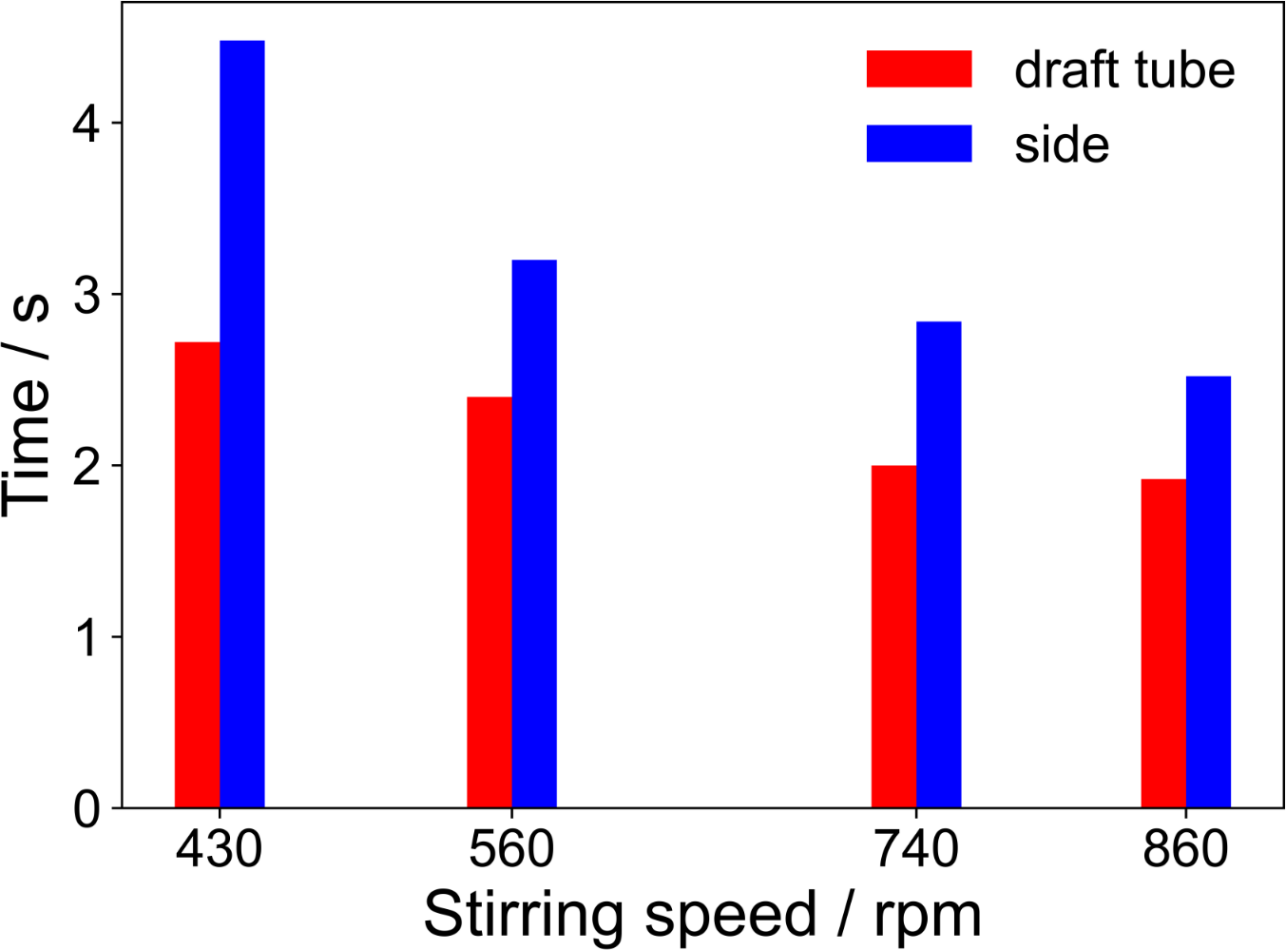
Quantified mixing time using different stirring speeds.

Figure 5 shows the determined mixing times for the different regions. The mixing time in the draft tube ranged from 2.72 s to 1.92 s and the mixing time outside of the draft tube from 4.48 s to 2.52 s for 430 rpm and 860 rpm, respectively. The low dependency of the mixing time in the draft tube on the stirring speed might be related to the small size of the propellers used in the draft tube, which might interfere with the draft tube, finally reducing their effectiveness at higher stirring speeds. However, the propeller used below the draft tube is larger and will not suffer from interference with the draft tube, enhancing the convection outside the draft tube.

#### Photonic characterization

**Figure 6:**
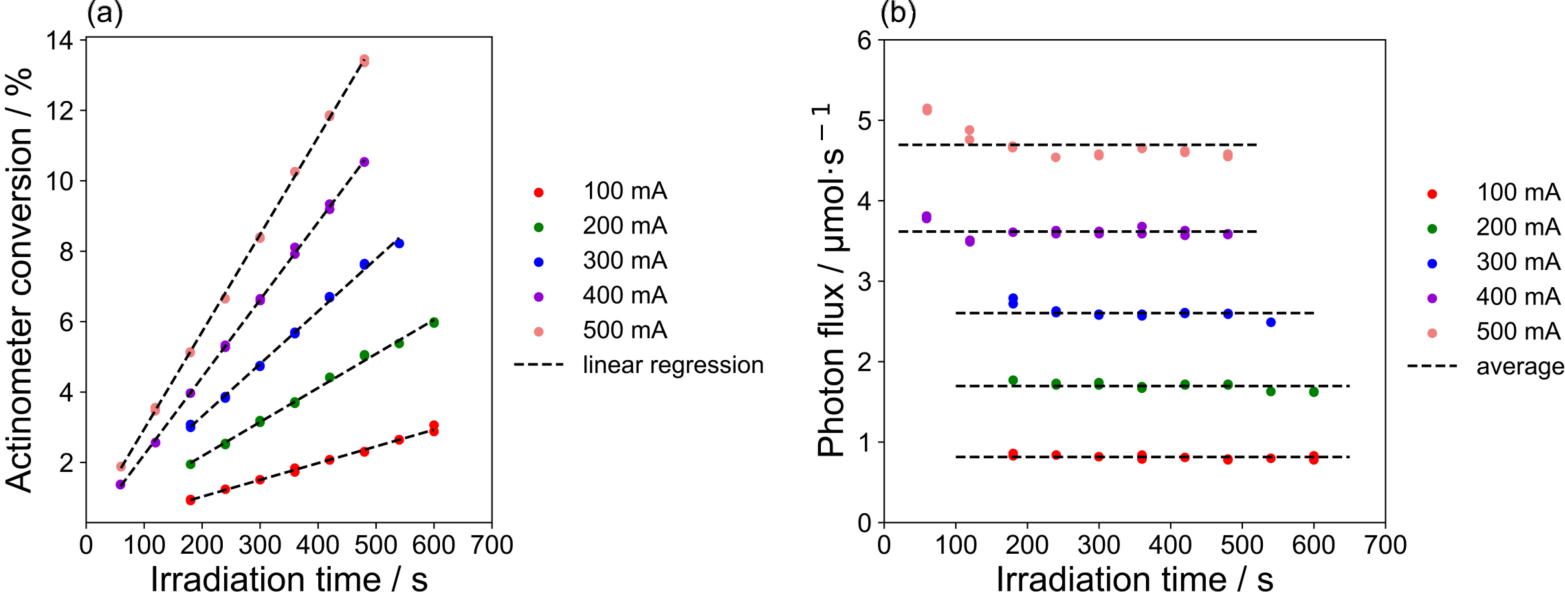
Chemical actinometry results: (a) actinometer conversion at different irradiation time, (b) photon flux at different irradiation time.

The loop photoreactor using 6 LEDs was photonically characterized by chemical actinometry. Figure 6 shows the actinometer conversions and calculated photon fluxes at different irradiation times. The reasonable linear fits of the conversion (see Figure 6a) prove the applicability of the chosen experimental protocol for the loop photoreactor [31]. Consequently, time-independent photon fluxes were calculated for the different operational conditions (see Figure 6b). Only for very short times in combination with high electrical currents of LEDs (e.g., 500 mA) a change of the photon flux is observed. For short times this is caused by very low conversions and the associated experimental uncertainties and for the latter conditions by the application limits of the ferrioxalate actinometer [32].

**Figure 7:**
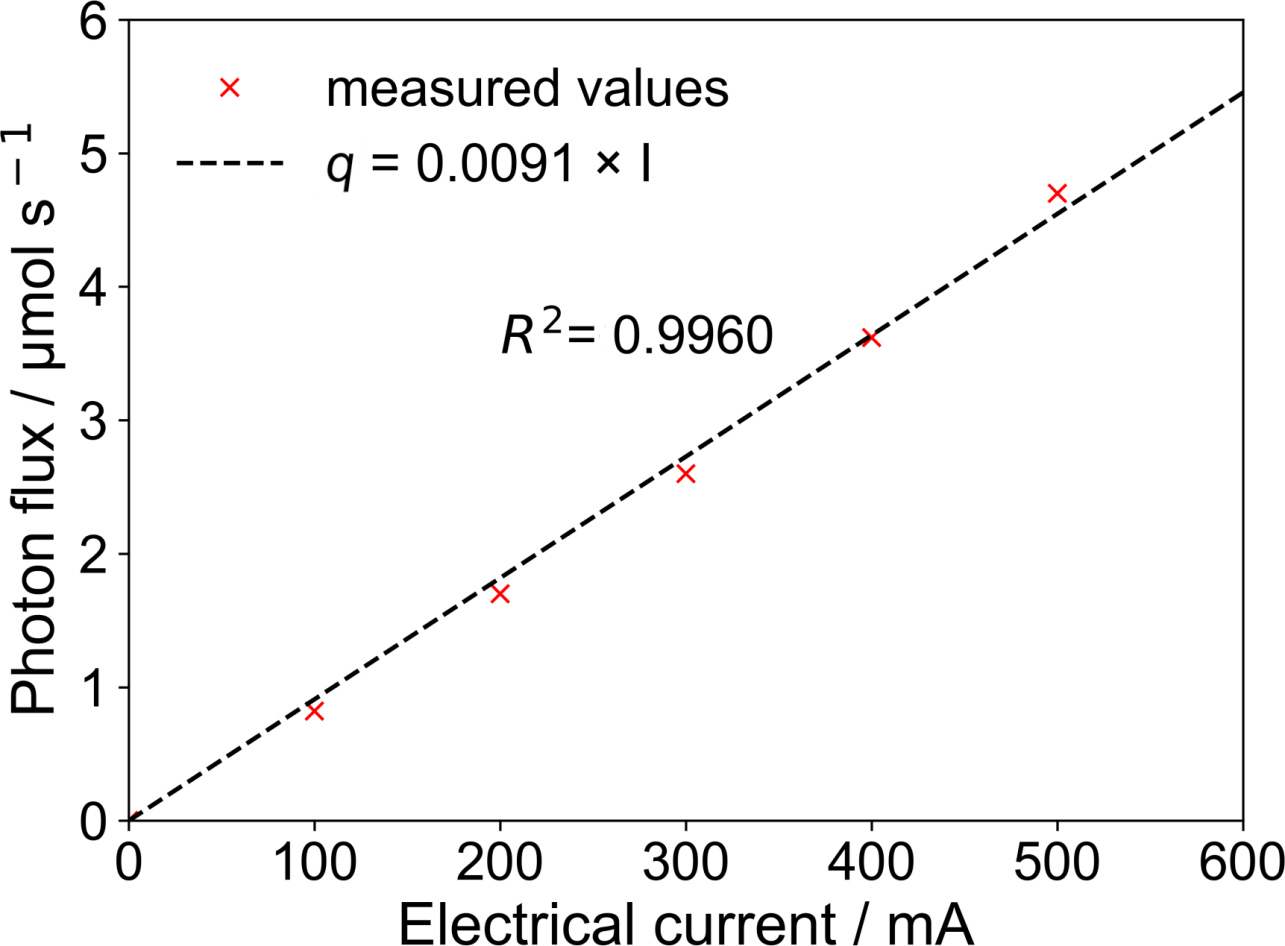
Photon flux in the loop reactor with respect to the LED electrical current.

The average photon flux incident on the reaction solution depends linearly on the LEDs electrical current (see Figure 7). The good linear regression shows that the chemical actinometry gave quite reliable photon flux values in the loop photoreactor. This relation was used to extrapolate the photon fluxes for experiments with more than 6 LEDs or with higher electrical current.

#### Light absorption

According to the Beer–Lambert law, the absorption of light in a solution can be described by Equation 1 [33].


**Figure 8:**
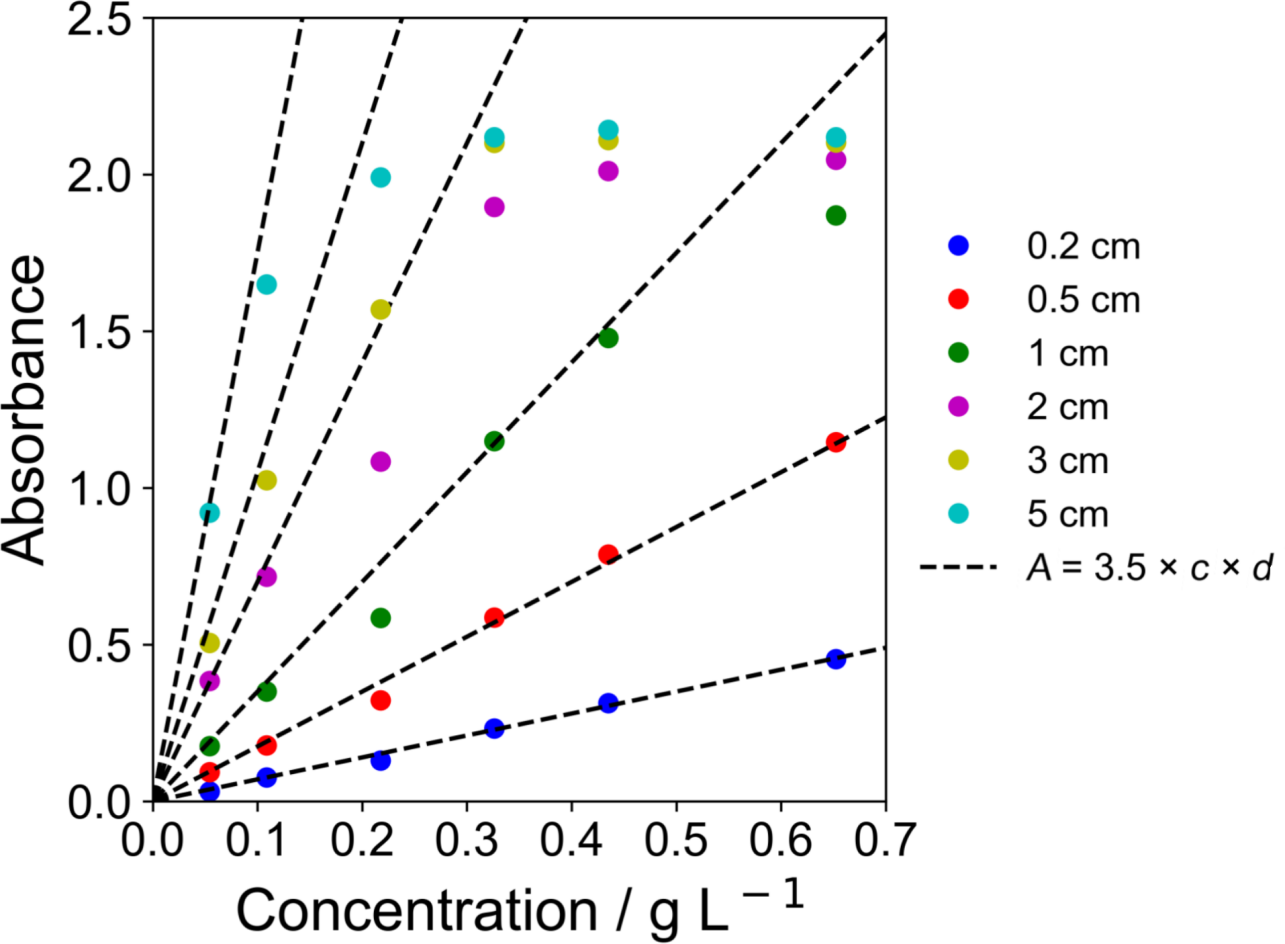
Absorbance as function of photocatalyst loading for different optical path lengths.

[1] $$\;A=\log_{10}\frac{I_{0}}{I_{\text{d}}}=\varepsilon \times c\times d$$

where *A* is the absorbance, *I*_0_ is the intensity of light entering the solution perpendicularly to one face, *I*_d_ is the intensity of light exiting the solution, ε is the extinction coefficient, *c* is the concentration of a light-absorbing solute, and *d* is the optical path length. Figure 8 shows the measured absorbance as function of the photocatalyst loadings in the methanol/water solution using different optical path lengths. The absorbance depends linearly on the photocatalyst concentration for low concentrations, which agrees with the Beer–Lambert law [33–34]. An extinction coefficient of 3.5 L g^−1^ cm^−1^ was determined by fitting the experimental data. The deviation of the absorbance from the linear relation is attributed to increased scattering at high loadings and/or long optical paths, where consequently the requirements for the application of the Beer–Lambert law are not fulfilled anymore. Considering the inner diameter of the loop reactor of 5 cm, a catalyst loading of 0.11 g L^−1^ is sufficient to absorb more than 95% of the photons.

### Photocatalytic hydrogen evolution

#### Long-term operation

Photocatalytic hydrogen evolution was performed using water and methanol under UV LED irradiation with a Pt-PCN photocatalyst (R1). The volume ratio of water and methanol used in this work is 9:1 with a total volume of 460 mL. The used setup was equipped with a micro-GC for online measurements of the hydrogen evolution, which enabled sampling every 3 minutes and thus provides insights on the kinetics of hydrogen evolution. Compared to conventional manual sampling with gas-tight syringes, online measurements also improve reproducibility by minimizing potential errors such as contamination by air or sampling mistakes.

**Figure 9:**
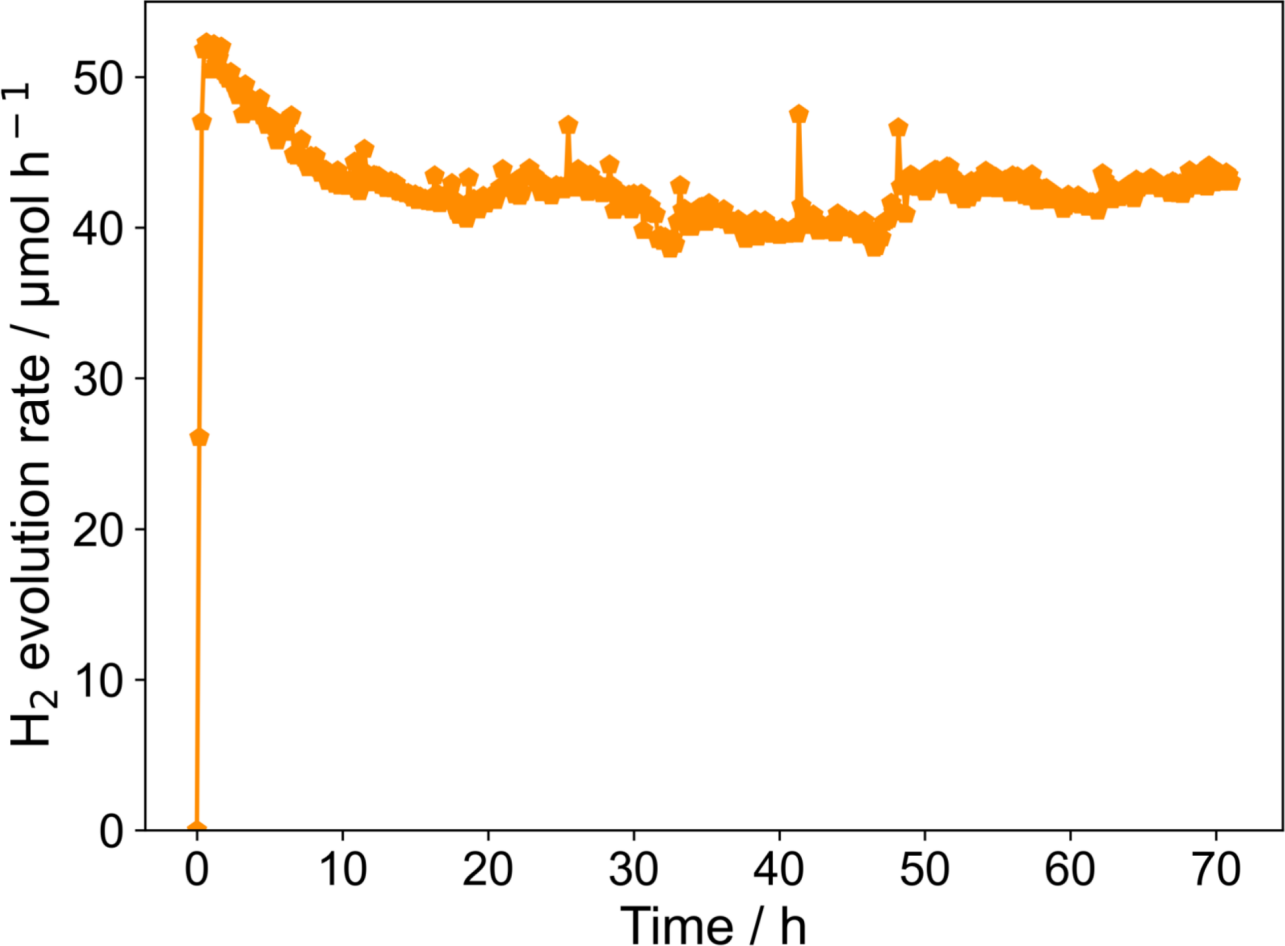
Long-term operation of the loop photoreactor.

The long-term stability of the photocatalyst was initially tested in the loop photoreactor with a photocatalyst loading of 0.22 g L^−1^, a stirring speed of 560 rpm, a photon flux of 4.70 μmol s^−1^, and an inert gas flow rate of 75 mL min^−1^. Figure 9 shows the hydrogen generation rate as function of irradiation time. The hydrogen generation rate first increases to a maximum value and decreases subsequently. After around 10 hours of irradiation, a steady level of hydrogen evolution is reached. Even though some fluctuations in the range of ±8% can be observed, the hydrogen evolution rate remained at this level for 70 hours of irradiation. These results prove the long-term stability of the systems, which is a prerequisite for any future application.

#### Design of experiment analysis

During design of experiment (DOE) analysis, the default linear and quadratic model were found to describe the experimental data reasonably well (see Table 1). While R^2^ is higher for the quadratic model, it must be checked whether the model is over-parameterized with terms that are not statistically significant. To do this, the R^2^_adj_ and R^2^_pred_ were analyzed. The R^2^_adj_ plateaus when insignificant terms are added to the model, and R^2^_pred_ will decrease when there are too many insignificant terms. The low R^2^_pred_ value of 0.6213 confirms that the default quadratic model is over-parameterized. For a reasonable model, the difference of R^2^_adj_ and R^2^_pred_ should be within approximately 0.2 [35]. As it is evident from Table 1, this is the case for the linear (0.0831) but not for the quadratic mode one (0.3033). Therefore, compared to the default quadratic model, the linear model is more appropriate for predicting maximum hydrogen generation rate within the value range of the studied parameters.

**Table 1:** Performance metric values for the suggested models from DOE analysis.

Performance metric	Model		

	Linear	Quadratic	Modified

R^2^	0.8619	0.9686	0.9526
R^2^_adj_	0.8343	0.9246	0.9289
R^2^_pred_	0.7512	0.6213	0.8426
R^2^_adj_ – R^2^_pred_	0.0831	0.3033	0.0863

The significance of the chosen parameters was evaluated by analysis of variance (ANOVA). The “Prob>F” probability value gives the estimation of whether the parameter has a significant effect on the response, and generally a parameter with a probability value less than 0.05 is considered as significant. A probability value larger than 0.10 renders the parameter generally as insignificant [35]. It is evident from Table 2 that light intensity and inert gas flow can be regarded as significant, while the photocatalyst amount and the stirring speed are insignificant.

**Table 2:** “Prob>F” values of the studied parameters from DOE ANOVA analysis.

Parameter	Prob>F	

	Linear model	Modified quadratic model

*q*	<0.0001	<0.0001
*c*	0.1671	0.8276
*ν*	0.9278	0.1305
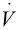	0.0028	<0.0001
*q* × 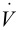	–	0.0068
*c* × *ν*	–	0.0279
*c* ^2^	–	0.0164
*ν* ^2^	–	0.0054

In addition to this analysis, it is necessary to check if there are any interactions between the studied parameters. After the insignificant parameter interaction terms were removed from the quadratic model, the interaction of photocatalyst loading and stirring speed has a “Prob>F” value of 0.0279, which means this interaction term also significantly affects the maximum hydrogen generation rate. The interaction effect was summarized in the modified quadratic model, which has a difference between R^2^_adj_ and R^2^_pred_ of 0.0863. Moreover, an ANOVA showed that the *p*-value of the linear and modified quadratic model is lower than 0.0001, which confirms that the model is adequate with a significance level of more than 99%. Therefore, the linear and modified quadratic models are suitable for describing the relation between photocatalytic hydrogen generation and the four studied parameters.

The linear and modified model from DOE analysis are described with Equation 2 and Equation 3:


[2] $$PR=-13.92+6.49\;q+15.70\;c+1.07\times 10^{-3}v+0.42\dot{V}$$

**Figure 10:**
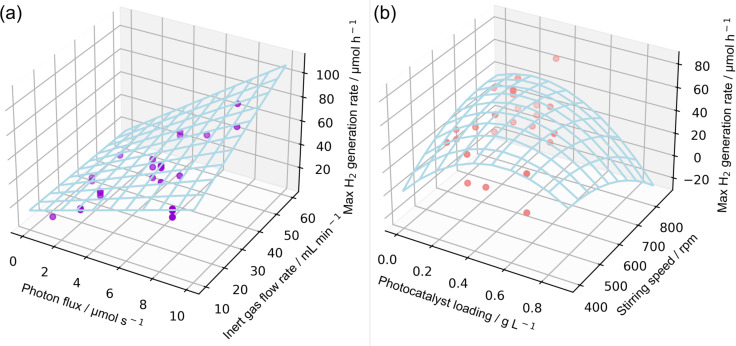
Visualization of the response surfaces of the modified DOE model for the four parameters: (a) photon flux and inert gas flow rate, (b) photocatalyst loading and stirring speed.

[3] $$\begin{array}{c} PR=-150.23+3.31\;q+255.36\;c+0.38\;v-7.77\times 10^{-3}\dot{V}\\ +\;0.1\;q\times \dot{V}-0.22\;c\times v-149.86\;c^{2}-2.42\times 10^{-4}v^{2} \end{array}$$

where *PR* represents the predicted maximum photocatalytic hydrogen generation rate (µmol h^−1^), *q* is the photon flux (µmol s^−1^), *c* is the photocatalyst loading (g L^−1^), *v* is the stirring speed (rpm), and 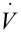
is the inert gas flow rate (mL min^−1^). The response surfaces for the modified model are shown in Figure 10. Linear correlations dominate the response to the photon flux and inert gas flow rate, while the power law correlations dominate the photocatalyst loading and the stirring speed.

**Figure 11:**
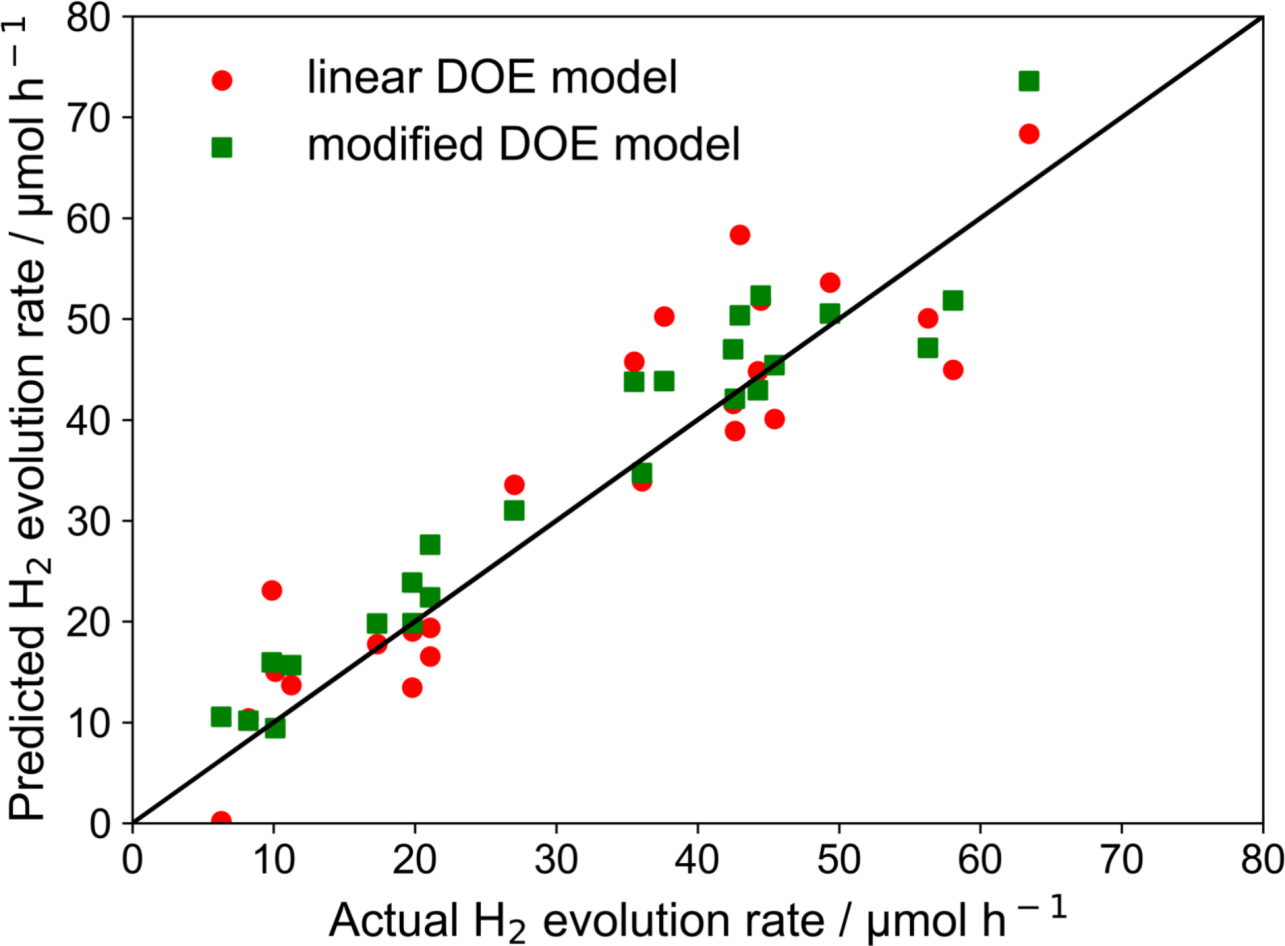
Parity plot of the measured photocatalytic hydrogen generation rate versus the values predicted by the models from DOE analysis.

Figure 11 shows that a reasonable parity between the predicted hydrogen generation rate and the actual experimental hydrogen generation rate could be achieved. No significant difference between the prediction accuracy of the two models is observable. The only difference between the two models is that the modified model considers the interaction of parameters. Therefore, to validate the model from DOE analysis, experiments that vary only the individual parameters were performed to see which model is more appropriate.

#### Analysis of individual reaction parameters

**General considerations:** To test the validity of the model derived with the DOE approach, the capabilities of the model prediction were tested against conventional single parameter studies. For that, additional experiments were conducted and analyzed, when possible, together with the data points from the DOE study. Given the significance of the photon flux and the inert gas flow rate, these parameters were studied first. To mimic the conventional experimental approach, the photocatalyst loading and the stirrer speed were investigated as well.

**Figure 12:**
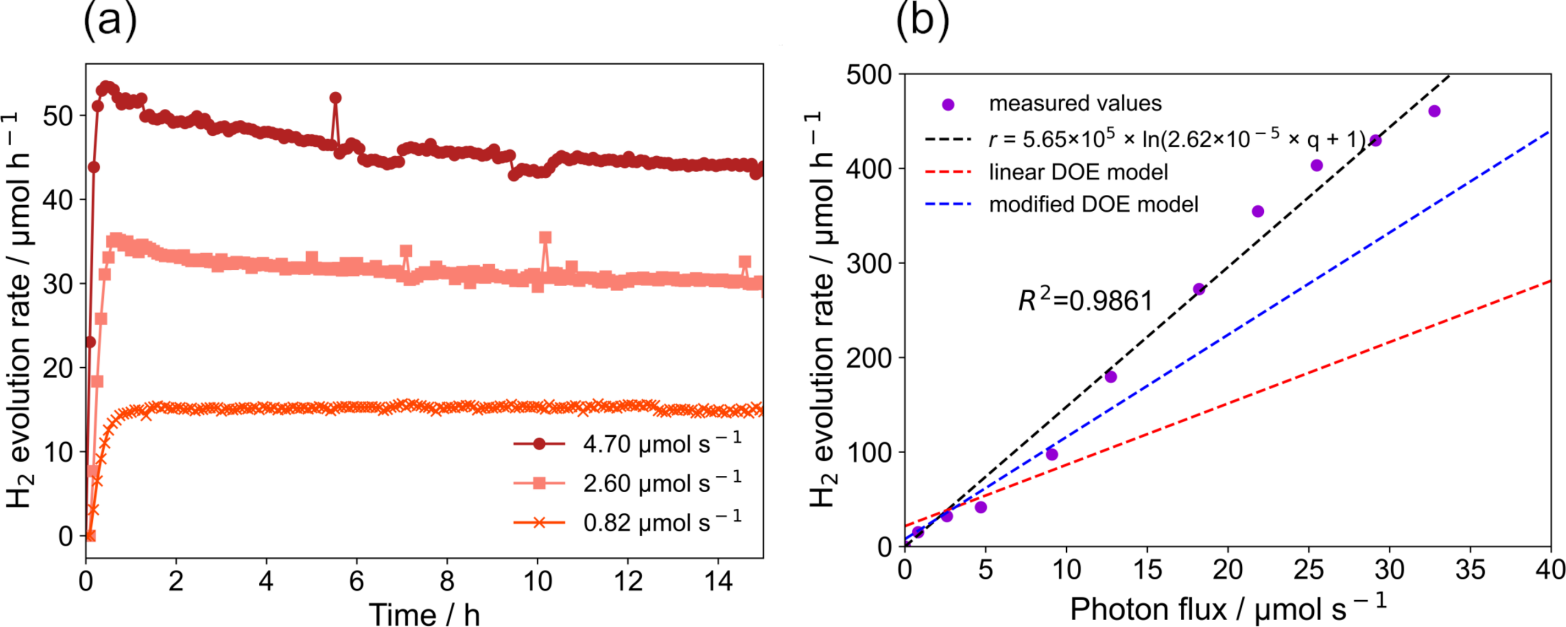
Effect of photon flux on hydrogen generation rate: (a) hydrogen generation rate as function of the irradiation time under different light intensities, (b) hydrogen generation rate as function of the photon flux.

**Photon flux:** The effect of the photon flux on the temporal evolution of the hydrogen generation rate was investigated individually for a constant set of operating parameters (*c* = 0.22 g L^−1^, *v* = 560 rpm, 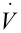 = 75 mL min^−1^). The photon flux related to the LEDs’ electrical current is calculated based on the actinometry results presented in section "Photonic characterization" (see Figure 7). The characteristics of the temporal evolution of the hydrogen generation rate were found to depend on the photon flux (Figure 12a). For all conditions, the observed hydrogen evolution rate increased initially. At a low photon flux, a steady state evolution rate was reached quickly and remained stable during the reaction. However, at higher photon fluxes, the hydrogen generation rate decreased slowly with the irradiation time (up to 18% after 15 hours for a photon flux of 4.70 µmol s^−1^).

An increase of the maximum hydrogen generation rate with an increase of the incident photon flux was found. The maximum hydrogen generation rate was 3.5 times higher for a 5 times higher photon flux of 4.70 µmol s^−1^ than at a photon flux of 0.82 µmol s^−1^ [36]. Furthermore, it was observed that an increasing photon flux reduced the time needed for reaching the maximum hydrogen generation rate. Nomikos et al. reasoned a similar observation with a decreased time needed for complete reforming of methanol and reaction intermediates at higher intensities [37].

For further studies, the reactor setup was equipped with four heat sinks around the reactor, each equipped with 6 LEDs. This was the constructive maximum of LEDs that could be installed and allowed for providing a maximum photon flux of 32.77 µmol s^−1^ to the reactor. Systematic studies on varying photon fluxes showed an almost linear increase of the hydrogen evolution rate with lower photon fluxes (see Figure 12b).

Generally, three regimes can be differentiated for the correlation of the reaction rate with the photon flux [37–42]. At low photon fluxes (regime 1), the hydrogen generation rate is typically limited by the available photon flux and can be approximated first order with respect to light intensity. For higher photon fluxes, kinetic limitations appear (second regime) since the larger amount of charge carriers present in the photocatalysts causes accelerated recombination in parts of the reactor. When extremely high photon fluxes are used, the reaction would be kinetically limited at every point in the reactor (regime 3). Increasing the photon flux would not increase the reaction rate anymore. Bloh proposed a model linking the photon flux and reaction rate [42], which was used in this work to fit the experiment results (see Equation 4).


[4] $$\langle r\rangle =\frac{k^{*}\times \theta }{\alpha }\times \ln\left(\frac{\phi \times q_{\text{p}}\times \alpha \times c_{0}}{k^{*}\times \theta \times c_{0}\times k_{\text{r}}}+1\right)$$

where 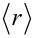 is the average (observed) reaction rate, k^*^ is the kinetic rate constant, θ is the surface coverage of the photocatalyst, *α* is the optical density, ϕ is the quantum yield, *q*_p_ is the volumetric photon flux density, *c*_0_ is the photocatalyst concentration, and *k*_r_ is the recombination rate.

The linear correlation between reaction rate and photon flux visible in Figure 12b indicates that the system is still in regime 1 and the hydrogen generation rate is limited by the available photon flux, even for the highest photon flux used in this study. The predictions from the DOE analysis hold for low photon fluxes but the inaccuracy increases with the photon flux. The deviations to the experimental data are larger for the linear as for the modified DOE model. For instance, the linear model predicts an about 50% lower hydrogen evolution rate for a photon flux of about 35 µmol s^−1^.

**Figure 13:**
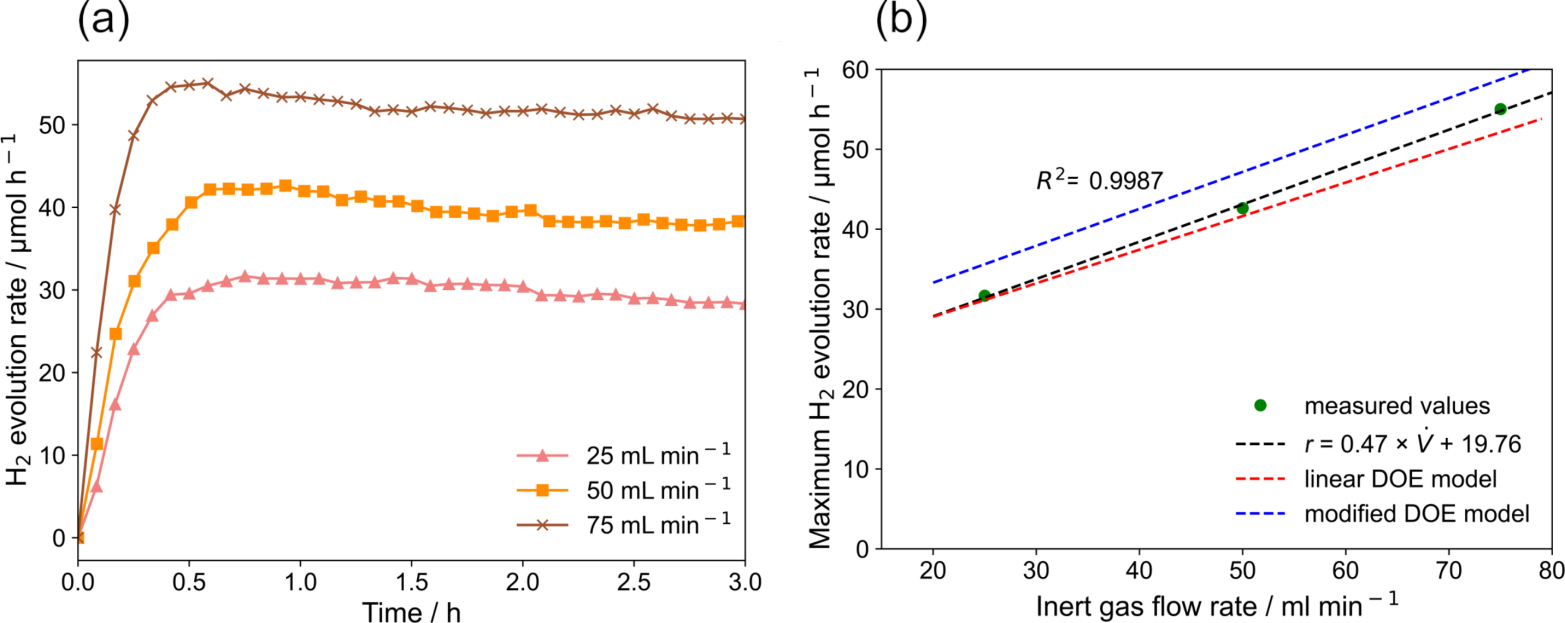
Effect of inert gas flow rate on hydrogen generation rate: (a) hydrogen generation rate as a function of the irradiation time, (b) maximum hydrogen generation rate as a function of the inert gas flow rate in the solution.

**Inert gas flow rate:** The effect of inert gas flow rate on hydrogen generation rate is shown in Figure 13a (*q* = 4.70 µmol s^−1^, *c* = 0.22 g L^−1^, *v* = 560 rpm). The maximum hydrogen generation rate increased by 74% when the inert gas flow rate was increased from 25 to 75 mL min^−1^. This effect is attributed to an enhanced mass transfer of hydrogen from the photocatalyst to the gas phase. The relevant mass transport steps for hydrogen include the diffusion from the photocatalyst surface to the liquid bulk, transfer from the liquid bulk to liquid–gas interface, and transfer from liquid–gas interface to gas phase [43]. Since the inert gas was purged into the reactor continuously, the generated hydrogen could also directly be transferred from the photocatalyst surface to the gas phase if a gas bubble got in contact with the catalyst surface. Furthermore, it is evident from Figure 13a that higher inert gas flow rates decrease the irradiation time needed to reach the maximum hydrogen generation rate. At a flow rate of 25 mL min^−1^ it took around 45 min to reach to maximum hydrogen generation rate, while at a flow rate of 75 mL min^−1^ only around 30 min was sufficient. Besides, the decrease of the activity with reaction time is less pronounced compared to the studies on the influence of the photon flux. Both observations are also attributed to the enhancement of mass transfer induced by a higher inert gas flow rate.

The maximum hydrogen generation rate is linearly correlated to the inert gas flow rate (see Figure 13b). This is in accordance with the mass transfer studies published by Escudero et al. [43], who also showed that the hydrogen generation rate is linearly related to the gas flow rate. Moreover, the linear relationship is also supported by the DOE analysis. Both DOE models predict the experiment results quite well. The linear model yields a slightly better prediction. A linear regression of only the data points shown here shows an expected good agreement with the experimental data.

**Figure 14:**
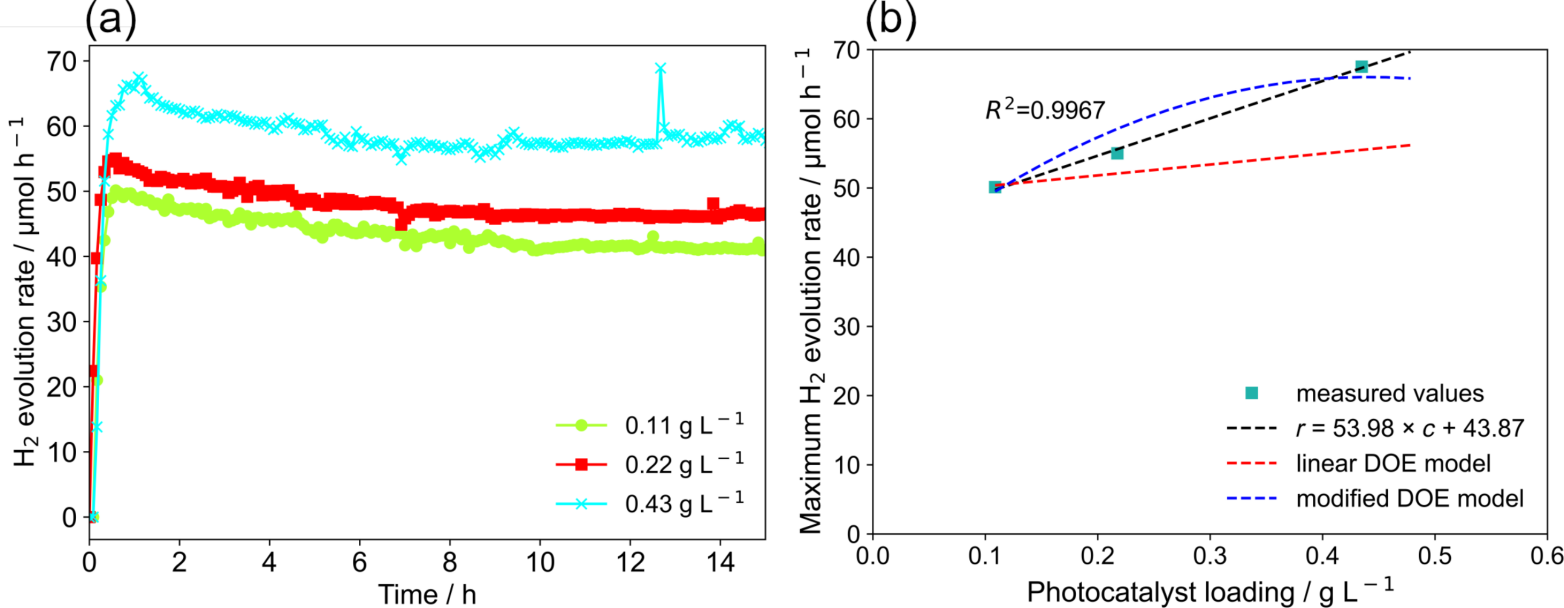
Effect of photocatalyst loading on the hydrogen generation rate: (a) hydrogen generation rate as function of irradiation time, (b) maximum hydrogen generation rate as function of photocatalyst concentration.

**Photocatalyst loading:** The temporal hydrogen evolution activity for different photocatalyst loadings is shown in Figure 14a (*q* = 4.70 µmol s^−1^, *v* = 560 rpm, 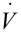
= 75 mL min^−1^). Again, the hydrogen generation rate increased to a maximum and then slowly decreased until a steady state rate was reached, likely due to the same reasons as discussed above. An increase of the photocatalyst loading enhanced the hydrogen generation rate, which might be a result of improved overall light harvesting at higher loadings and indicates that the mass transport issues (i.e., transport of the reactants to and of the products away from the surface catalytic sites) is still not rate-limiting in this regime [38].

Figure 14b shows the predicted maximum hydrogen generation rate for varying photocatalyst loadings. It is obvious that the modified model deviates less from the experimental results. Despite this, it also becomes clear that the quadratic model used for the modified model is not suited for extrapolations since a decrease of the hydrogen evolution activity is predicted which is not found experimentally. In fact, a linear model, that is fitted solely to this small data set and ignores the effect of the other reaction parameters, yields a better prediction (see Figure 14b). This would also be in line with observations reported by Nomikos et al. for a 0.5 wt % Pt/TiO_2_ photocatalyst suspended in water and methanol solution at low loadings (from 0 to 0.33 g L^−1^) [37]. These authors found a saturation point at higher photocatalyst loadings after which hydrogen generation activity did not increase any further.

**Figure 15:**
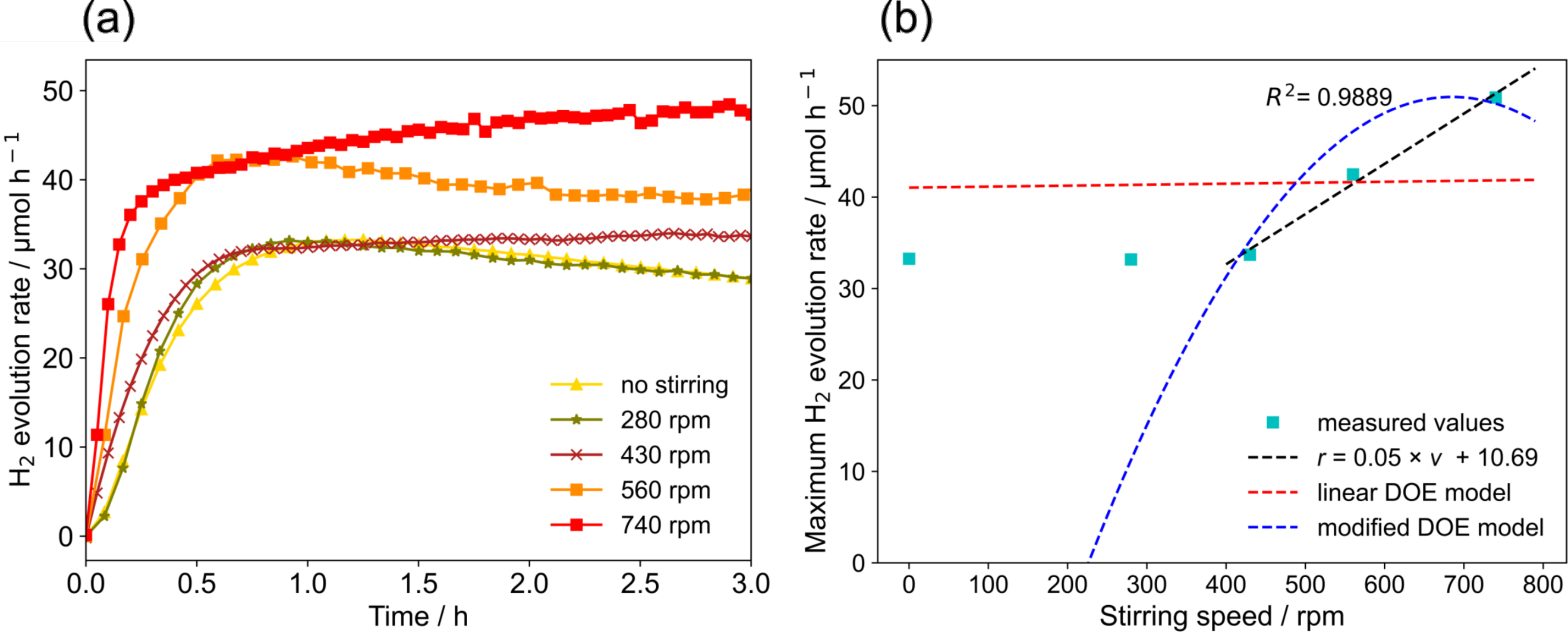
Effect of stirring speed on hydrogen generation rate: (a) hydrogen generation rate as function of irradiation time, (b) maximum hydrogen generation rate as function of stirring speed.

**Stirring speed:**Figure 15a shows the hydrogen evolution activity for different stirring speeds (*q* = 4.70 µmol s^−1^, *c* = 0.22 g L^−1^, 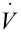 = 50 mL min^−1^). Increasing the stirring speed results in an increase in the maximum hydrogen generation rate. The maximum hydrogen generation rate was 1.5 times higher at 740 rpm than at 430 rpm. This gives evidence that increased convection improves the mass transfer of generated hydrogen from the liquid phase to the gas phase or even directly from the catalyst to the gas phase. This is in line with the measured mixing time. Furthermore, the experiments show that a certain threshold is required to achieve higher activity. Only for stirring speeds above 430 rpm, an improvement is observed. This is most likely due to limited convection at lower stirring speeds and the hydrodynamic issues discussed above.

Figure 15b shows the prediction of the maximum hydrogen generation rate for different stirring speeds. The modified DOE model shows a better agreement with the experimental results for conditions above 280 rpm than the linear model, but again the physicochemical plausibility of the quadratic model is questionable. It is evident from the shown data that a linear fit describes the experimental data well when the correlations with other parameters and the data points for “no stirring” and a stirring speed of 280 rpm are excluded. The required threshold for achieving sufficient convection is likely the reason why a linear description cannot be applied for the data set.

**Discussion:** Analysis of the single parameter studies in comparison to the DOE analysis shows that the studied parameter set possesses complex correlations that cannot be described in the required quality with the simple mathematical models available for the DOE analysis. With power law models the description of the experimental data can be improved in terms of statistics, but physical and chemical plausibility cannot be ensured with this approach. Thus, only interpolations in a predefined parameter space are possible. In conclusion, application of simple statistical models to reduce the experimental efforts is not suited for the studied photocatalytic processes. The non-linear interplay of various parameters limits the value of the derived DOE models.

Independent from this statistical analysis, it is possible to compare the performance of the loop photoreactor and the suspended photocatalysts to results presented in the literature. The apparent quantum efficiency (AQE) is a suited performance metric for such a comparison and defined as [41]:


[5] $$\Phi_{\text{app}}=\frac{n\times r}{q}$$

where *n* is the number of electrons involved in the photocatalytic reaction (2 in this case), *r* is the hydrogen generation rate, and *q* is the total photon flux entering the reaction volume.

**Figure 16:**
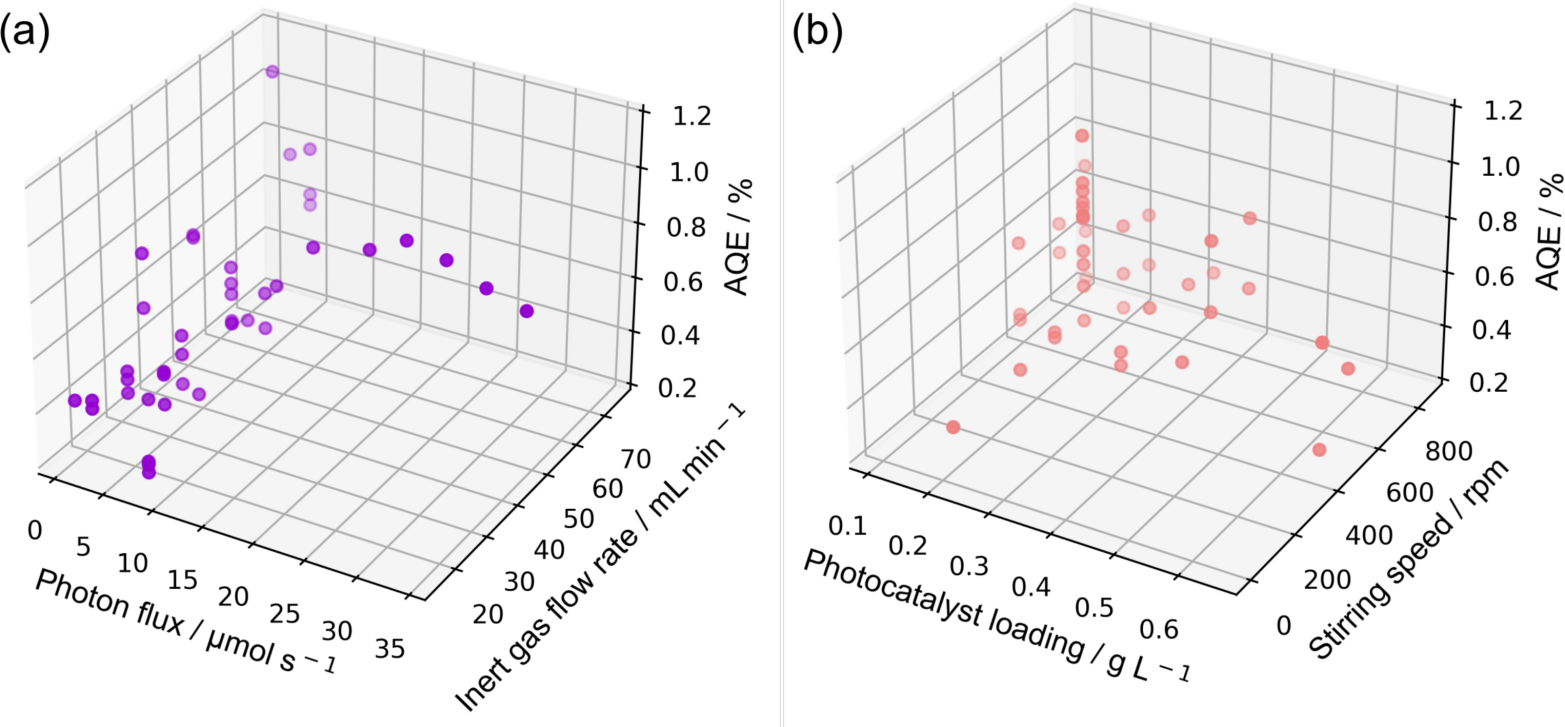
3D plot of AQE for different studied parameters: (a) AQE for different photon fluxes and inert gas flow rates, (b) AQE for differrent photocatalyst loadings and stirring speeds.

Figure 16 depicts the AQE at 365 nm calculated for all experimental data points. The determined AQE ranges from 0.25 to 1.05%, which is a factor of 4 to 17 higher as reported in the literature for the photocatalytic hydrogen evolution using Pt loaded polymeric carbon nitride in a 21 mL Schlenk tube (AQE = 0.06%) [30]. The maximum hydrogen generation rate observed was 460.66 µmol h^−1^ at a photon flux of 32.77 µmol s^−1^, a catalyst loading of 0.22 g L^−1^, a stirring speed of 560 rpm and an inert gas flow rate of 50 mL min^−1^. Under these conditions, an AQE of 0.75% was determined. The highest AQE of 1.05% was found for the lowest photon flux of 0.82 µmol s^−1^ (*c* = 0.22 g L^−1^, *v* = 560 rpm, 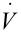 = 75 mL min^−1^). With respect to a high overall performance of the system, it is notable that even for high photon fluxes of more than 12 µmol s^−1^ AQE larger than 0.75% are observed. This indicates that the performance is not decreasing due to an increased rate of recombination, which is in line with the analysis of the photon flux dependency. Overall, the loop photoreactor not only allows for an order of magnitude increase in reaction scale, but also significantly increases the efficiency of light utilization.

## Conclusion

The presented investigations show that loop photoreactors are suited to scale up the light-driven hydrogen evolution with Pt-loaded polymeric carbon nitride photocatalysts. With the presented design, a scale-up by more than one order of magnitude could be realized compared to literature-reported systems. Systematic evaluation of operational parameters revealed a significant influence of the photon flux as well as the inert gas flow rate. The latter finding indicates that sufficiently fast mass transport is of major importance for the performance of the studied reaction. The used loop reactor provides excellent mass transport properties, which eventually enabled an increase of the apparent quantum yield by a factor of 10. This result is even more relevant than the demonstration of the scale-up as efficient use of photons provided to the reaction solution is the basis for a future large-scale application of solar-driven hydrogen evolution.

The findings emphasize that an interdisciplinary investigation of light-driven reactions is of utmost importance for implementing photocatalytic hydrogen generation on a large scale. The strong interaction of the radiation field with mass transport of the involved species, i.e., light absorber, catalyst, reactants, and hydrogen as product, has a tremendous effect on the overall performance. Engineering aspects may have direct implications for the design of photocatalytic materials to cope, e.g., with mass transport issues on a larger scale. Consequently, reaction engineering studies should not only be conducted after a catalytic system was developed but should be an integral part of research on light-driven systems already during development of photocatalytic active materials.

## Experimental

### Reactor design

The design parameters for the loop photoreactor are based on the optimal design parameters suggested by Blenke et al. [44]. The draft tube diameter is adjusted to achieve maximal mass transfer in the reactor [45–48]. The design parameters used in this work are defined by Equations 6–9:


[6] $$D_{\text{D}}=\;0.44\;\times D_{\text{R}}$$

[7] $$L_{\text{D}}=\;4.4\;\times D_{\text{R}}$$

[8] $$L_{\text{T}}=\text{ 0}.4\;\times D_{\text{R}}$$

[9] $$L_{B}=\text{ 0}.4\;\times D_{\text{R}}$$

where *D*_R_ is the inner diameter of the reactor, *D*_D_ is the inner diameter of the draft tube, *L*_D_ is the draft tube length, *L*_T_ is the distance of the draft tube to the reactor top, *L*_B_ is the distance of the reactor bottom to the draft tube. The reactor is manufactured from 2 mm thick borosilicate glass with a total height of 40 cm. Borosilicate glass was chosen as the reactor body material based on the following reasons: (1) the transmission of borosilicate glass at UV range is already over 90%, (2) borosilicate glass is easier to work with for manufacturing the reactor with the proposed design, and (3) borosilicate glass is the cheaper material. With 2 mm thickness, about 10% of the incident light is absorbed by the reactor wall. The inner diameter of the reactor *D*_R_ is set as 5 cm, yielding a total reactor volume of around 500 mL.

**Figure 17:**
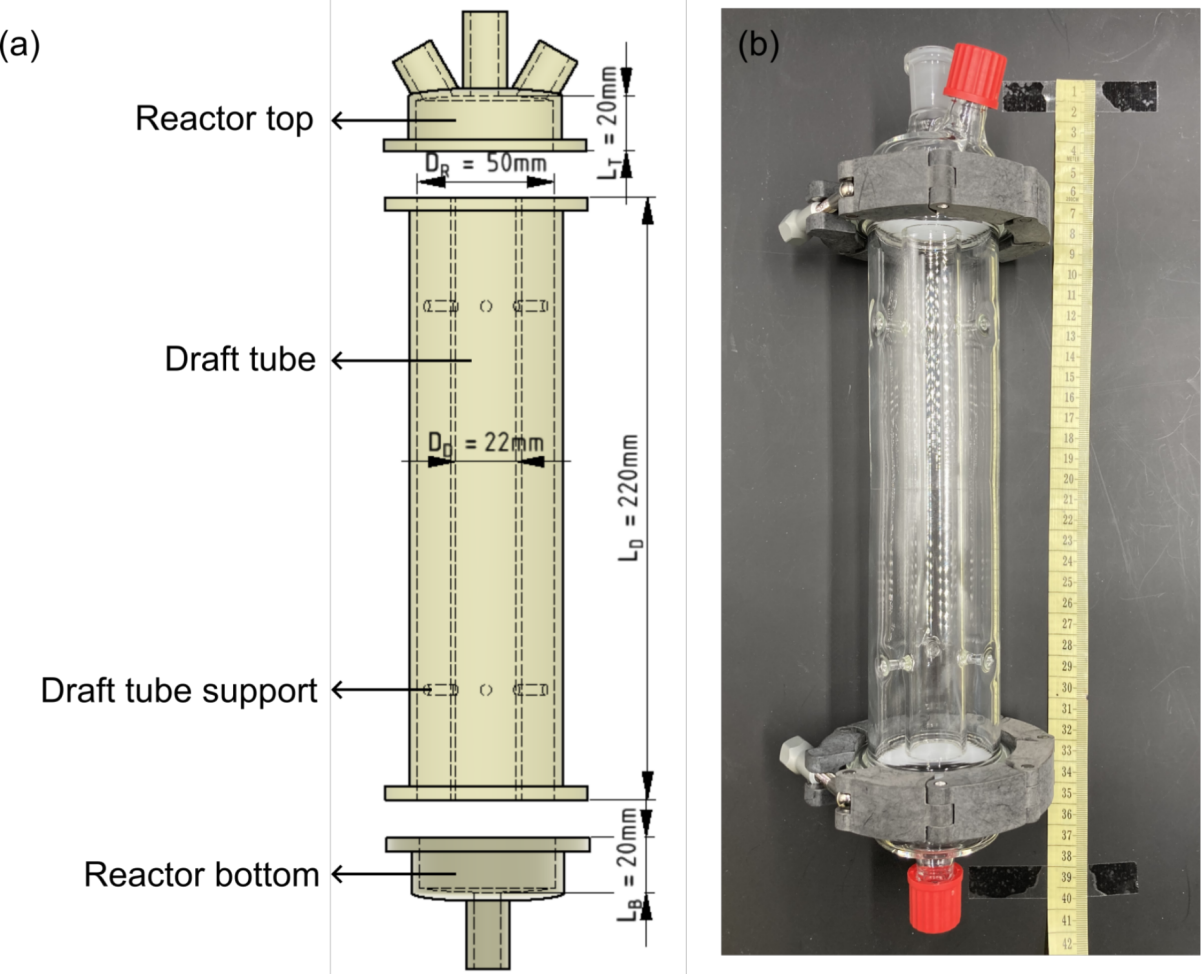
Reactor design: (a) CAD drawing of the loop reactor, (b) picture of the manufactured loop reactor.

The designed loop photoreactor consists of three separate parts, the reactor head with a gas inlet and a gas outlet (GL18 thread), the reactor body with draft tube, and the reactor bottom (see Figure 17a). The three parts are assembled using two clamps (plastic black, DN 50) and O-rings. The reactor top has a 29/32 ground joint for an overhead stirrer.

**Figure 18:**
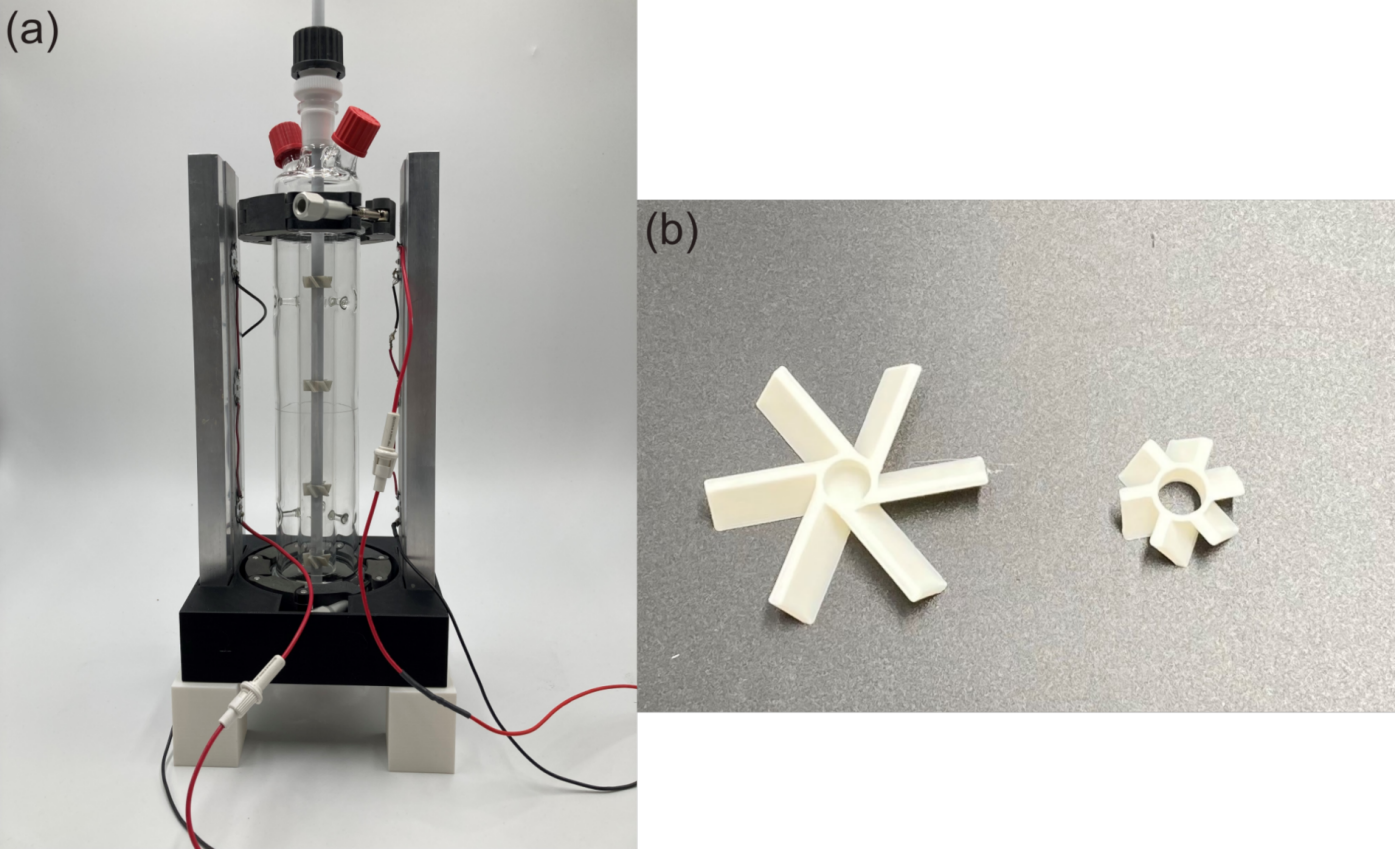
Assembled reactor and 3D printed parts: (a) the whole reactor setup, (b) 3D printed propellers.

3D-printed support structures were used to ensure a reproducible installation for the reactor setup (see Figure 18a). The propellers used to induce the required convection in the draft tube were also 3D-printed. One large propeller was mounted at the bottom of the shaft tube and four small propellers were mounted on the shaft above the big propeller (see Figure 18b). The mounted position of the propeller on the shaft is shown in Supporting Information File 1, Figure S2a. To ensure that the propellers were fixed at the same position on the shaft tube, holes with M3 thread were drilled in the shaft. All 3D-printed parts were printed with a 3D printer (Pro2 Plus, Raise3D) using a polylactic acid (PLA) filament. The 3D printer setting is shown in Supporting Information File 1, Table S1.

As light source 365 nm UV LEDs (Luminus SST-10-UV, Luminus Devices, Inc.) were used. The technical specification of the UV LED is shown in Supporting Information File 1, Table S2. If not mentioned specifically, a total number of 6 LEDs was used, which were mounted on 2 plate-fin heat sinks (each heat sink was equipped with 3 LEDs). The position of the LEDs on the heat sink is shown in Supporting Information File 1, Figure S2b. The design of reactor support and propellers are shown in Supporting Information File 1, Figures S3 and S4.

### Reactor characterization

The fluid flow pattern in the loop photoreactor was experimentally studied with a tracer experiment. The reactor was initially filled with 460 mL deionized water. The stirrer motor was then turned on with an appropriate rotational speed. When a steady hydrodynamic state was reached in the reactor, 5 mL of a 0.8 g L^‒1^ methylene blue solution was injected at the top surface of water at the top of the reactor. To ensure reproducibility and comparability, the methylene blue solution was always rapidly injected at the same spot using a syringe with a large opening. The tracer injection time was kept constant throughout the experiment at 1.2 s. The coloring of the solution was monitored with a video camera (Sony ZV-1). The video was shot under red light, which matched the absorption properties of the dye, to obtain optimal optical visualization. Digital images extracted from videos (see Supporting Information File 2) were processed with the Python OpenCV package to quantify the mixing time (see Supporting Information File 3, Python code) by following the color evolution in the videos from red towards black under the red-light environment [49]. ROI used as the working zone were defined on the images when these were processed to quantify the mixing time. The three rectangles shown in Figure 3 on frame at 1.2 s showed the regions used for image processing. To quantify the mixing time, the RGB color model was used, representing each color as a mixture of pure red, green, and blue light of varying levels ranging from 0 to 255 [50]. Because the videos were shot under red light, only the red channel values were used for mixing time quantification. For each frame of the video, the average red channel value of all the pixels in the marked ROIs were calculated. The mixing time calculation is explained in Supporting Information File 1 and the Python code for the corresponding calculation is shown in Supporting Information File 3.

The loop photoreactor was photonically characterized by chemical actinometry using a ferrioxalate actinometer [31,51]. For each measurement, 495 mL 0.04 M K_3_Fe(C_2_O_4_)_3_ solution was irradiated with 365 nm LEDs (6 in total, mounted on 2 heat sinks). Then different electrical currents of LEDs are used to get the corresponding photon flux in the reactor. Depending on the electrical current used for the experiment, the irradiation time was in a range of 1–10 min (conversion between 1 and 13%). From the conversion, the incident photon flux was calculated.

### Photocatalyst synthesis and characterization

For the synthesis of the photocatalyst, melamine (99%, Sigma-Aldrich), H_2_PtCl_6_·6H_2_O (with a minimum Pt content of 37.50%, Sigma-Aldrich), and methanol (99.9%, VWR) were utilized. The heptazine-based polymeric carbon nitride (PCN) was synthesized by thermally condensing 30 g of melamine at 550 °C for 4 h with a heating rate of 10 °C min^−1^. The resulting yellow solid was then finely grounded using an agate mortar. For the photochemical deposition of the Pt co-catalyst, 6 g of the produced PCN were first dispersed in 340 mL of water and subjected to sonication for 90 min. Subsequently, a mixture containing 40 mL of methanol and 20 mL of an H_2_PtCl_6_·6H_2_O solution (0.03 mM) was added to the dispersion. The resulting suspension was stirred under N_2_ atmosphere for 45 min followed by irradiation over 2 h with six 365 nm LEDs (with a total photon flux of 4.7 µmol s^−1^) mounted on 2 heat sinks (see Supporting Information File 1, Figure S2b), while maintaining a continuous flow of N_2_ in the headspace through a septum. The resulting suspension exhibited a yellow/greenish color and was subsequently washed with deionized water 3–5 times, followed by drying at 70 °C overnight and grinding. The synthesized photocatalyst is referred to as Pt-PCN.

The surface morphology, chemical composition, and elemental distribution of the Pt-PCN were analyzed using a ZEISS LEO 1550 VP scanning electron microscope (SEM) equipped with energy dispersive spectroscopy (EDS) from Ametek, USA. The SEM was operated at an acceleration voltage of 15 kV. To prepare the sample for SEM imaging, a few milligrams of the Pt-PCN material were dispersed in 1.0 mL of ethanol using sonication for 5 min. Subsequently, a thin film of the sample was obtained by drop-casting a few microliters of the suspended sample onto a conductive Cu foil substrate and allowed to dry at room temperature. To improve the conductivity and achieve higher resolution SEM imaging, a 20 nm thick layer of Au was sputtered onto the sample surface. For EDS mapping, the samples were not sputtered with Au to avoid interference during the determination of Pt.

### UV–vis electronic absorption measurement

Light attenuation in the reaction solution was measured for different optical paths (0.2, 0.5, 1, 2, 3, and 5 cm) with the same concentration of photocatalyst in water and methanol solution as used for photocatalytic hydrogen evolution. Cuvettes were used to model the reactor system. The cuvette was first put in a self-designed cuvette holder covered with cuvette holder cover (see CAD drawing of 1 cm light path cuvette holder and cuvette holder cover design as an example in Supporting Information File 1, Figures S5 and S6) with collimators (COL-UV/VIS, Avantes). All the 3D models for cuvette holder and cuvette holder cover used for different light paths are provided in Supporting Information File 4. Two Avantes fiber cables (FC-UV-600-2-BX and FC-UVIR600-2-ME) were used for the absorbance measurement. The light source used was an Avantes light source (AVALIGHT-DHC s/n: LS-2001019) and an Avantes spectrometer (AvaSpec-Mini2048CL) was used to measure the absorbance with AvaSoft software. An example of the whole absorption measurement setup using 1cm light-path cuvette and the cuvette holder setup is shown in Supporting Information File 1, Figure S7.

### Photocatalytic hydrogen evolution

**Figure 19:**
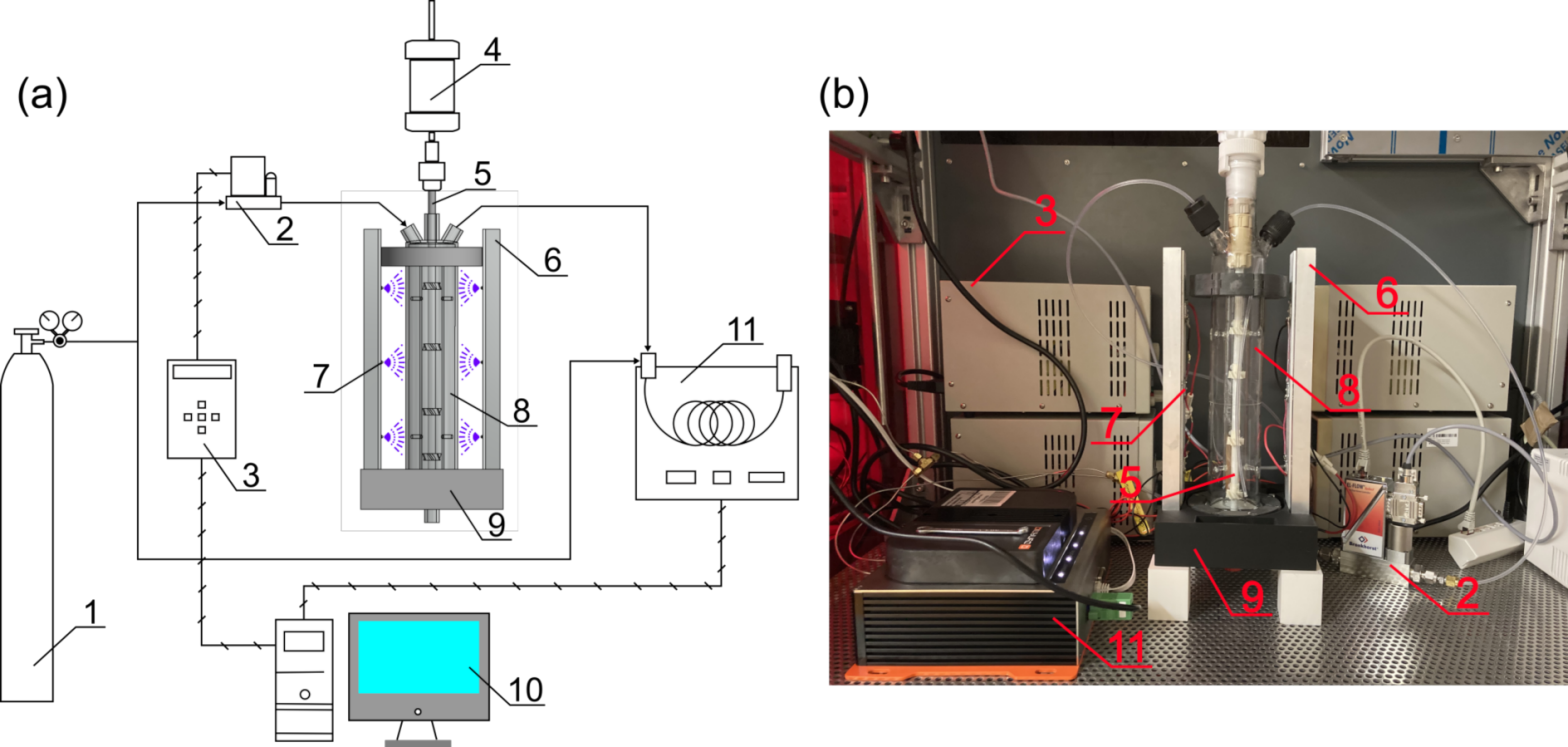
Photocatalytic reaction setup: (a) reaction setup flow diagram, (b) reaction setup in lab. 1 argon gas cylinder, 2 mass flow controller (MFC), 3 MFC controller, 4 overhead stirrer motor, 5 shaft tube with propellers, 6 heat sink for LEDs, 7 LED, 8 loop photoreactor, 9 3D-printed support structures, 10 computer, and 11 micro GC.

The flow diagram of the setup used for photocatalytic hydrogen evolution is shown in Figure 19. The photocatalyst particles were first put into 414 mL water and dispersed by ultrasonication. The suspension was then filled into the loop photoreactor together with 46 mL methanol, adding up to a total of 460 mL solution in the reactor (methanol/water = 1:9, v/v). The reactor was then sealed and the overhead stirrer motor (HS-D, Witeg Labortechnik GmbH) was turned on for stirring with the magnetic stirrer (Magnetic Stirrer Head (P-MRK), BOLA). Argon gas served both as carrier gas for the used micro GC (DynamiQ-S, Qmicro B.V.) and as inert gas to remove the oxygen in the reactor. Before the light source was turned on, argon gas was purged into the solution to remove dissolved oxygen. During the reaction, a constant argon flow was set with a mass flow controller (MFC, F-201CV-2K0-RGD-33-V, Bronkhorst High-Tech B.V.). The light source was then turned on and the photocatalytic reaction was allowed to proceed. The outlet of the reactor was connected to the GC as online analytics, probing the gas composition in 3 min intervals. The product gas stream was allowed to vent into an exhaust gas line. To align the results using photocatalysts from different batches, an adjustment factor of 2.212 was calculated. The calculations are shown in Supporting Information File 1, Figure S8.

### Design of experiments

A DOE analysis was used to evaluate the effect of four operating parameters, i.e., photon flux *q*, photocatalyst load *c*, stirring speed *v*, and inert gas flow rate 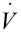 on maximum photocatalytic hydrogen generation rate. These selected parameters were considered as the independent variables and the maximum hydrogen generation rate (µmol h^−1^) was selected as the dependent variable (response). Design-Expert^®^ software (10.0.5.0 Stat-Ease, Inc.) was used to perform the DOE analysis. To avoid random experimental values from a range of chosen values of the parameters when using the software, the values for the independent variables were chosen manually based on the technical properties of the reaction setup, which are listed in Table 3. The studied DOE set of experiments with different combination of parameters is shown in Supporting Information File 1, Table S3. A total of 25 parameter combinations for the four independent variables was used for parameter analysis. Collected results were evaluated based on the coefficient of determination (*R*^2^), adjusted coefficient of determination (*R*^2^_adj_) and predicted coefficient of determination (*R*^2^_pred_). These coefficients were calculated based on Equations 10–12.


[10]

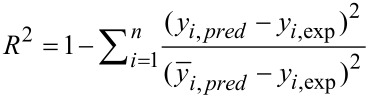




[11] $$R_{\text{adj}}^{2}=1-\left[\left(1-R^{2}\right)\frac{n-1}{n-K-1}\right]$$

[12]

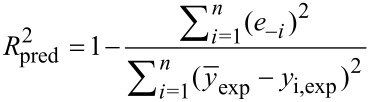



where *n* represents the number of data points, *K* is the number of input variables, *y*_i,pred_ is the predicted response, *y*_i,exp_ is the experimental data, 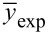 is the arithmetic mean of the experimental data, and *e_−i_* is a deletion residual computed by fitting a model without the *i*th run then predicting the *i*th observation with the resulting model used in the Design-Expert^®^ software statistical analysis.

**Table 3:** Parameter label and values used for design of experiments analysis.

Parameter	Label	Levels				

photon flux [μmol s^−1^]	*q*	0.82	2.60	4.70	6.37	8.19
photocatalyst loading [g L^−1^]	*c*	0.11	0.22	0.33	0.43	0.65
stirring speed [rpm]	*ν*	430	560	740	860	
inert gas flow rate [mL min^−1^]	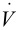	15	25	35	50	

## Supporting Information

File 1 Details of technical drawings, technical specifications, adjustment factor calculation, and DOE table.

File 2 Videos from fluid flow characterization.

File 3 Python code for video processing to get mixing time.

File 4 .stl files for 3D-printed parts and CAD drawings.

## Data Availability

The data that supports the findings of this study is available from the corresponding author upon reasonable request.
