# Supplementary material for: Optimizing reaction conditions for the light-driven hydrogen evolution in a loop photoreactor
Source: Beilstein J Org Chem. 2024 Jan 16;20:74–91. doi: 10.3762/bjoc.20.9 (PMC10804759; doi:10.3762/bjoc.20.9)
Supplement: File 1 — Details of technical drawings, technical specifications, adjustment factor calculation, and DOE table. [file Beilstein_J_Org_Chem-20-74-s001.pdf]

BEILSTEIN JOURNAL OF ORGANIC CHEMISTRY

Supporting Information

for

Optimizing reaction conditions for the light-driven hydrogen evolution in a loop photoreactor

Pengcheng Li, Daniel Kowalczyk, Johannes Liessem, Mohamed M. Elnagar, Dariusz Mitoraj, Radim Beranek and Dirk Ziegenbalg

Beilstein J. Org. Chem. doi:

Details of technical drawings, technical specifications, adjustment factor calculation, and DOE table

License and Terms: This is a supporting information file under the terms of the Creative Commons Attribution License (https://creativecommons.org/licenses/by/4.0). Please note that the reuse, redistribution and reproduction in particular requires that the author(s) and source are credited and that individual graphics may be subject to special legal provisions.
The license is subject to the *Beilstein Journal of Organic Chemistry* terms and conditions: (https://www.beilstein-journals.org/bjoc/terms)

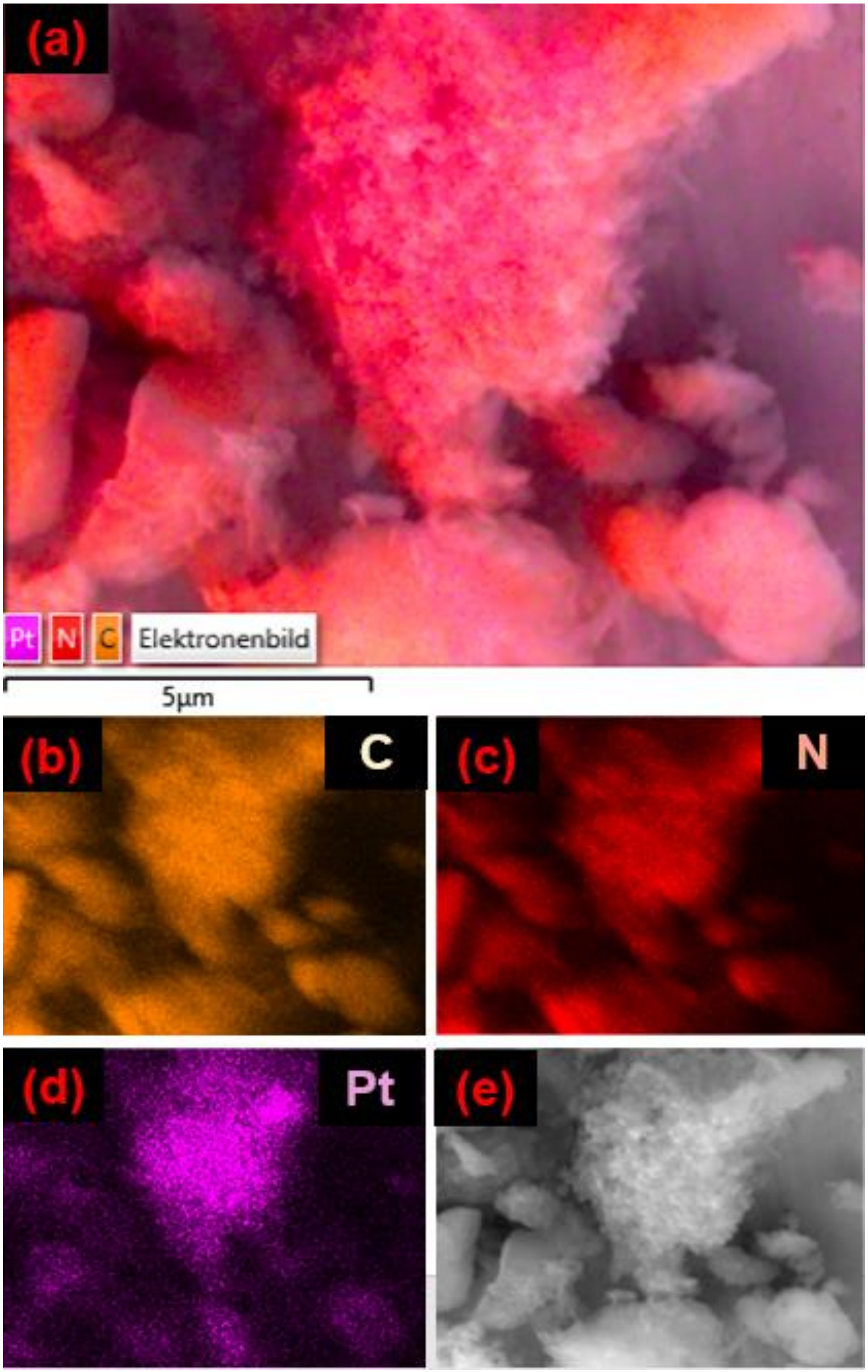

Figure S1. SEM-EDX elemental mapping images of C, N, and Pt on a particle.

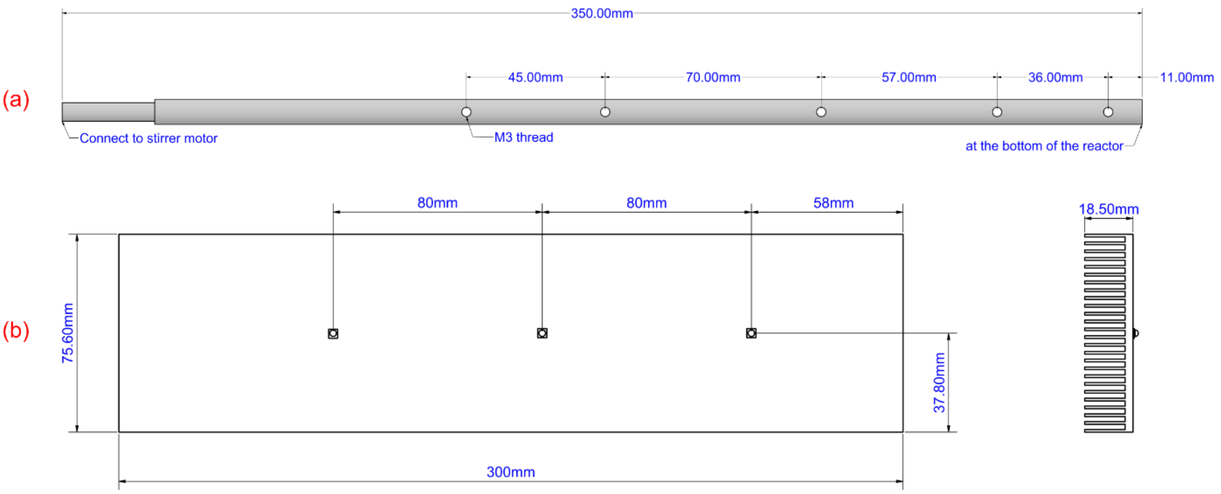

Figure S2. Geometry of (a) holes with M3 thread on shaft tube and (b) LEDs on heat sink.

Table S1. 3D printer setting for all 3D-printed parts.

Parameters	Magnitude	Unit
layer thickness	0.2	mm
infill density (%)	15	–
bed temperature	60	°C
extruder temperature	205	°C

Table S2. Technical specification of used UV LED (Luminus SST-10-UV, Luminus Devices, Inc.).

Parameter	Symbol	Values	Unit
peak wavelength range	λ	365–375	nm
	V_{Fmin}	3.0	V
rorward voltage	V_F	3.7	V
	V_{Fmax}	4.0	V
radiometric flux	Φ_{typ}	875	mW
absolute Maximum current	I_{max}	1.0	A
viewing angle	$2\varphi_{1/2}$	130	degrees

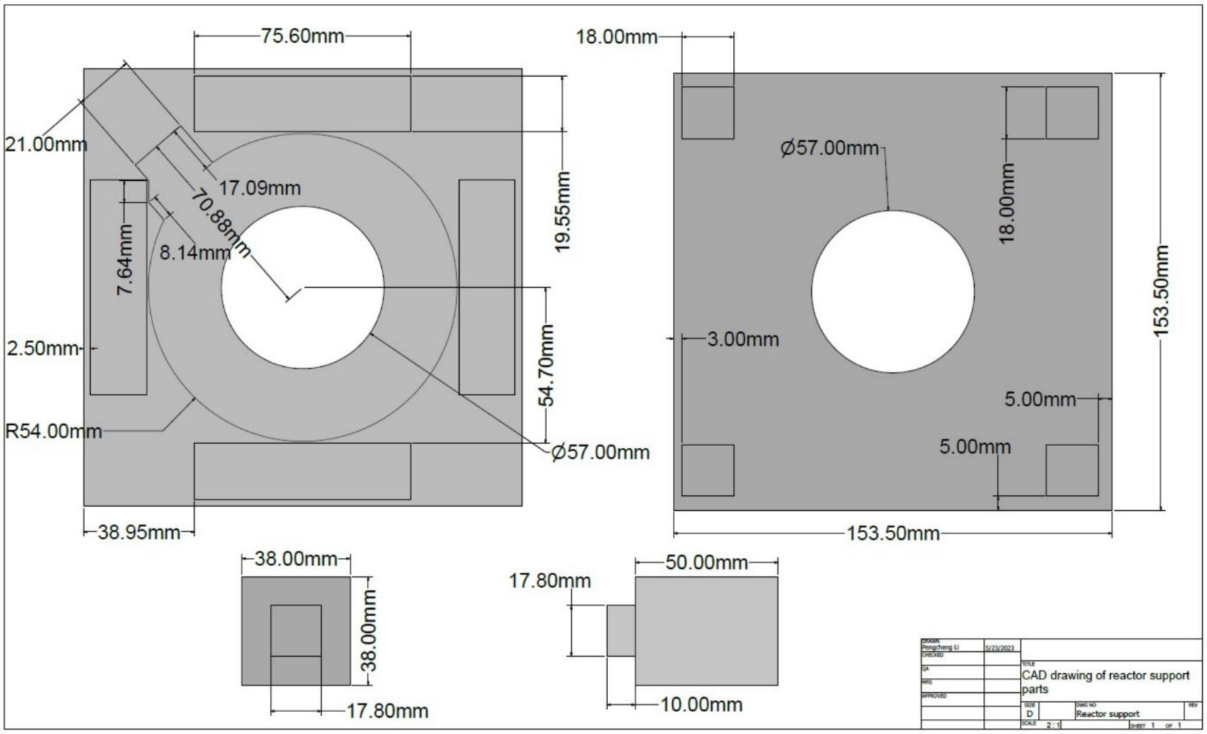

Figure S3. CAD drawing of reactor support parts.

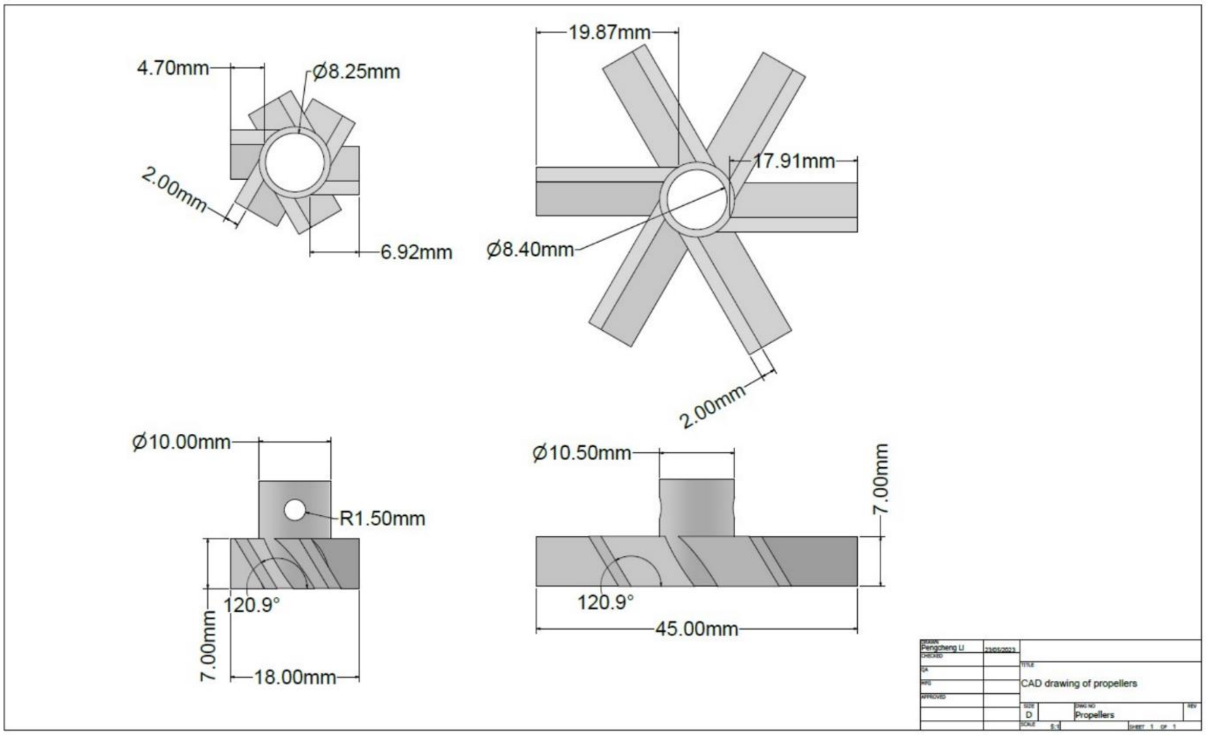

Figure S4. CAD drawing of designed propellers used for the experiments.

<u>Mixing time value calculation:</u>

To get the mixing time value, the difference of red channel values to the initial red channel value at the beginning of the experiment is calculated for different positions in the movie frames taken from the mixing experiments. Since the color tracer, a methylene blue solution, is blue and the reactor was illuminated with red light, the red channel value decreases in regions where the tracer is present. Therefore, the difference of red channel value would increase after the color tracer is injected. For each stirring speed, when the difference of red channel value is first larger than a threshold value (based on the red channel value plot), the corresponding time value is then extracted. For example, at stirring speed 430 rpm (Figure 4 in the publication), the difference of red channel values to the initial red channel value in the draft tube is calculated. After the color tracer is

injected at the top of the reactor, the difference increases. When the difference becomes larger than 4, it means the tracer is present. The time point when the difference first gets bigger than 4 is then extracted, which is at t = 1.36 s. The difference increases before it becomes constant, which means the mixing in draft tube is complete. This threshold value is 181.4, which corresponds to the time at t = 4.08 s. Then the mixing time in the draft tube at 430 rpm can be calculated, which is 2.72 s. The Python code for getting all mixing time is shown in Supporting Information File 3.

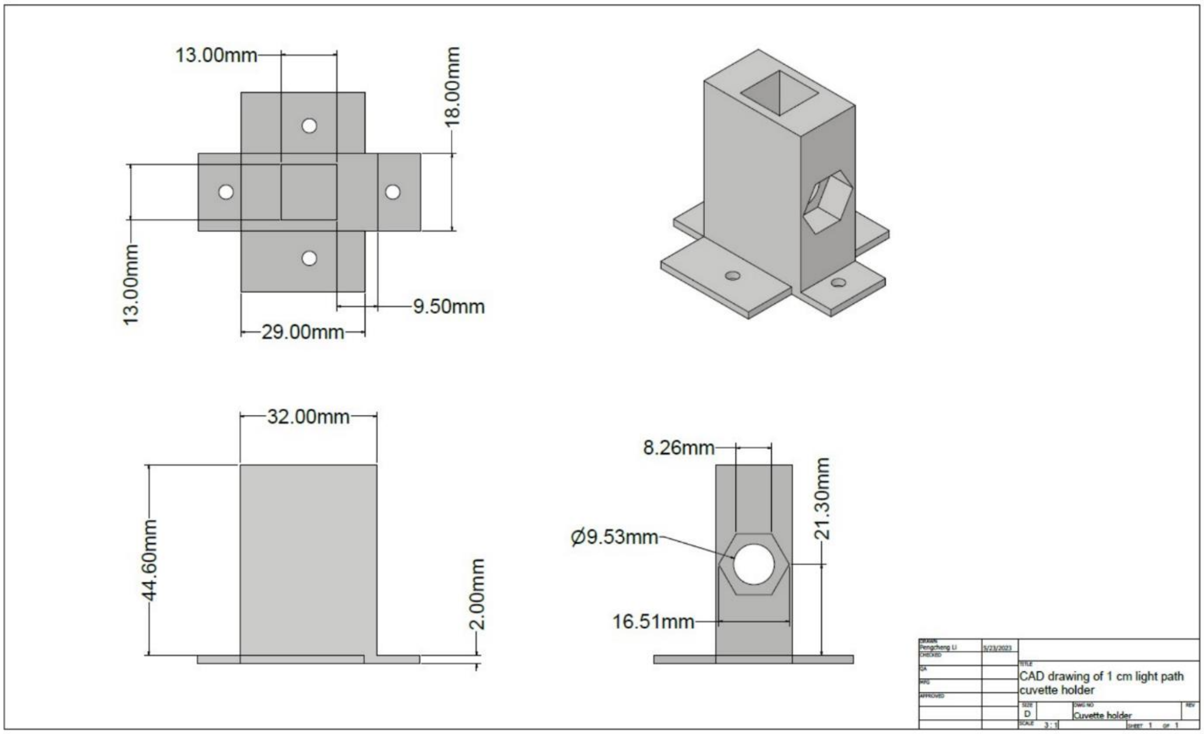

Figure S5. CAD drawing of 1 cm light path cuvette holder for absorption measurement.

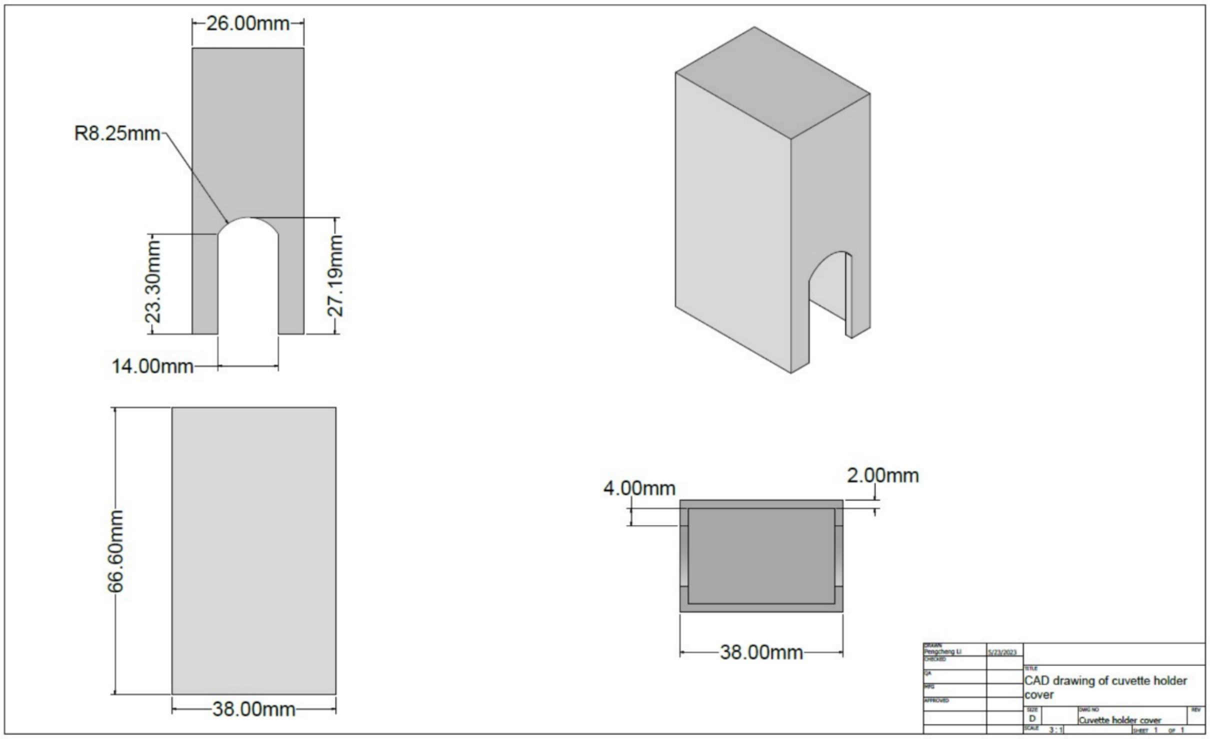

Figure S6. CAD drawing of 1 cm light patch cuvette holder cover.

The .stl files for reactor support, propellers, and all used holders can be found in Supporting Information File 4.

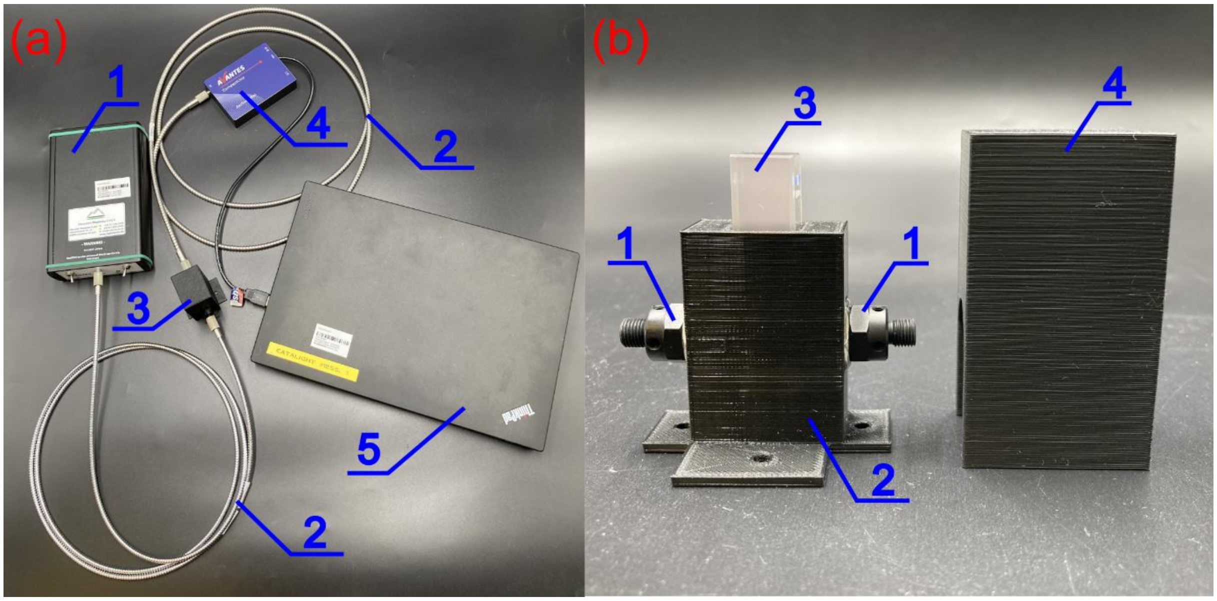

Figure S7. UV–vis absorption measurement setup: (a) whole absorption measurement setup: **1** light source, **2** optic fiber cable, **3** cuvette holder setup, **4** spectrometer, **5** computer; (b) cuvette holder and cuvette holder cover: **1** collimator, **2** cuvette holder, **3** cuvette, **4** cuvette holder cover.

Adjustment factor for results using photocatalysts from different batches:

Photocatalytic water reduction using photocatalysts from different batches was performed with the same operating condition (c = 0.22 g L^{-1}, r = 560 rpm, q = 4.70 μmol s^{-1}, $\dot{V}$ = 50 ml min^{-1}). Figure S8 shows that the hydrogen generation rate follows a similar behavior for photocatalysts from different batches. An adjustment factor was determined by calculating the mean hydrogen generation ratio of the entire irradiation time, which was 2.212. After applying the adjustment factor, the same hydrogen generation rate values could be achieved. Therefore, this adjustment factor was used to align the results from the two batches used.

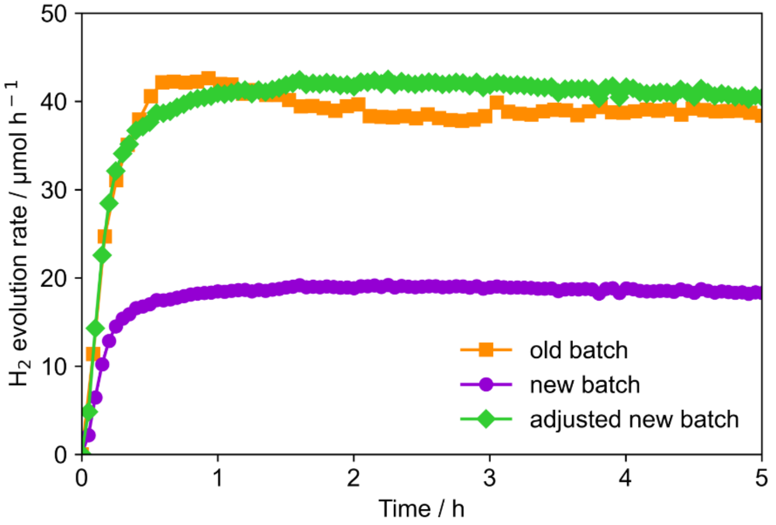

Figure S8. H_2 generation rate as a function of irradiation time using photocatalysts from different batches.

Table S3. Design of experiment with different combinations of parameters and their response values.

Run #	Independent variables				Response
	Photon flux / µmol s^{-1}	Photocatalyst loading / g L^{-1}	Stirring speed / rpm	Inert gas flow rate / ml min^{-1}	Maximum H_2 generation rate / µmol h^{-1}
1	0.82	0.22	860	35	8.21
2	4.70	0.11	560	35	27.03
3	0.82	0.11	860	50	10.11
4	6.37	0.33	560	25	58.07
5	8.19	0.33	860	50	63.44
6	2.60	0.33	740	25	21.10
7	2.60	0.22	740	15	19.80
8	6.37	0.22	430	50	51.81
9	8.19	0.43	560	50	82.64
10	4.70	0.11	740	50	45.43
11	2.60	0.22	860	25	17.32
12	6.37	0.65	560	25	56.30
13	4.70	0.11	860	35	36.06
14	6.37	0.33	430	25	44.26
15	4.70	0.43	740	35	42.63
16	4.70	0.22	560	50	42.49
17	0.82	0.11	740	15	6.28
18	0.82	0.65	430	50	9.87
19	8.19	0.65	430	15	42.98

20	8.19	0.22	740	15	44.46
21	6.37	0.11	560	35	35.50
22	2.60	0.43	430	15	21.06
23	8.19	0.11	860	15	37.63
24	2.60	0.33	430	25	19.82
25	0.82	0.43	740	35	11.24
