# Supplementary material for: Optimizing reaction conditions for the light-driven hydrogen evolution in a loop photoreactor
Source: Beilstein J Org Chem. 2024 Jan 16;20:74–91. doi: 10.3762/bjoc.20.9 (PMC10804759; doi:10.3762/bjoc.20.9)

Supporting Information File 3

Python script for video processing of mixing experiment

November 14, 2023

In [1]:

```
# Here are all the python packages used for video/image processing

import cv2
import numpy as np
import matplotlib.pyplot as plt
import pandas as pd
from scipy.optimize import curve_fit
import matplotlib.font_manager as fm
```

In [2]:

```
# Build video_process class which includes the necessary methods for video/image process

class video_process(object):
    def __init__(self, file_name):
        """
        :param file_name: the name of the video
        :type file_name: mp4

        """

        self.file_name = file_name

    def get_one_frame(self, frame_number):
        """
        Get the image of a frame based on the given frame number.

        :param frame_number: the frame number of the video you want to output
        :type frame_number: int

        :rtype: None

        """

        counter = 0
        video = cv2.VideoCapture(self.file_name)
        while video.isOpened():
            rete, frame = video.read()
            if rete:
                if counter == frame_number:
                    return frame
                counter += 1
            else:
                break
        video.release()
        return None

    def get_all_frames(self):
        """
        Get all frames of a video.

        :returns: a list of lists representing the frames from a video.
        :rtype: list

        """
```

```python
        video = cv2.VideoCapture(self.file_name)
        frame_list = []
        while video.isOpened():
            rete, frame = video.read()
            if rete:
                frame_list.append(frame)
            else:
                break
        video.release()
        return frame_list

    def get_region_avg(self, x_low, x_high, y_low, y_high):
        """
        Get the average red channel values of a marked region.

        :param x_low: the lower bound of x axis on the frame image
        :type x_low: int

        :param x_high: the higher bound of x axis on the frame image
        :type x_high: int

        :param y_low: the lower bound of y axis on the frame image
        :type y_low: int

        :param y_high: the higher bound of y axis on the frame image
        :type y_high: int

        :returns: the red channel value of the marked region
        :rtype: list

        """

        all_video_frames = self.get_all_frames()
        avg_red_vals = []
        for each_frame in all_video_frames:
            red_val = []
            for x in range(x_low, x_high):
                for y in range(y_low, y_high):
                    each_red_val = each_frame[y, x, 2]
                    red_val.append(each_red_val)
            sum_red = sum(red_val[:])
            avg_red = sum_red / len(red_val)
            avg_red_vals.append(avg_red)
        return avg_red_vals

    def get_time_line(self):
        """
        Get the time values of each frame in a video.

        :returns: the time values of frames in a video.
        :rtype: list

        """

        video_frames = self.get_all_frames()
        frame_counts = 0
        time_list = []
        video = cv2.VideoCapture(self.file_name)
        fps = video.get(cv2.CAP_PROP_FPS)
        for each_frame in video_frames:
            frame_counts += 1
            time_line = frame_counts / (fps) # Code for video from AG camera (Sony)
            time_list.append(time_line)
        return time_list
```

```
    def get_red(self):
        """
        Get the red channel values of the marked region used for the paper.
        Here are the x and y values on each frame image for ROI:
        dt vals: 130, 355, 10, 1750
        left vals: 30, 105, 10, 1750
        right vals: 370, 445, 10, 1750

        :returns: the red channel values of the marked region
        :rtype: list

        """

        draft_tube_vals = self.get_region_avg(130, 355, 10, 1750)
        left_side_vals = self.get_region_avg(30, 105, 10, 1750)
        right_side_vals = self.get_region_avg(370, 445, 10, 1750)
        return draft_tube_vals, left_side_vals, right_side_vals
```

Get frame images for Figure 7 in paper

In [3]:

```
video_560rpm = video_process(file_name="loop_560rpm.mp4")

# At t = 1.2 s

frame_560rpm_30 = video_560rpm.get_one_frame(30)
fix_frame_560rpm_30 = cv2.cvtColor(frame_560rpm_30, cv2.COLOR_BGR2RGB)

cv2.rectangle(fix_frame_560rpm_30, (30, 10), (105, 1750), color = (0, 255, 0), thickness
cv2.rectangle(fix_frame_560rpm_30, (130, 10), (355, 1750), color = (0, 255, 0), thicknes
cv2.rectangle(fix_frame_560rpm_30, (370, 10), (445, 1750), color = (0, 255, 0), thicknes
plt.imshow(fix_frame_560rpm_30)
plt.show()
```

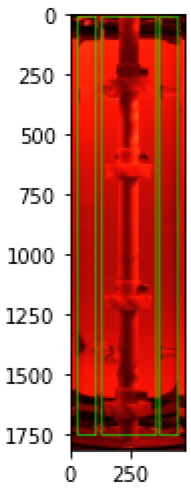

In [4]:

```
# At t = 2.2 s

frame_560rpm_55 = video_560rpm.get_one_frame(55)
fix_frame_560rpm_55 = cv2.cvtColor(frame_560rpm_55, cv2.COLOR_BGR2RGB)
plt.imshow(fix_frame_560rpm_55)
plt.show()
```

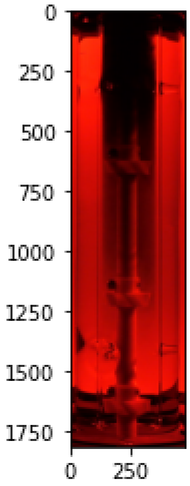

In [5]:

```
# At t = 4.2 s

frame_560rpm_105 = video_560rpm.get_one_frame(105)
fix_frame_560rpm_105 = cv2.cvtColor(frame_560rpm_105, cv2.COLOR_BGR2RGB)
plt.imshow(fix_frame_560rpm_105)
plt.show()
```

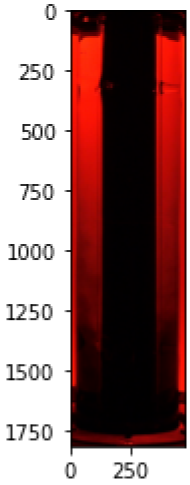

In [6]:

```
# At t = 7.8 s

frame_560rpm_195 = video_560rpm.get_one_frame(195)
fix_frame_560rpm_195 = cv2.cvtColor(frame_560rpm_195, cv2.COLOR_BGR2RGB)
plt.imshow(fix_frame_560rpm_195)
plt.show()
```

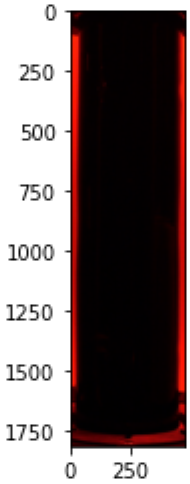

Get plot for Figure 8 in paper

In [7]:
```
# Get red channel values for stirring speed 430 rpm

video_430rpm = video_process(file_name="loop_430rpm.mp4")

dt_val_430rpm, left_val_430rpm, right_val_430rpm = video_430rpm.get_red()
dt_val_430rpm = np.asarray(dt_val_430rpm)
left_val_430rpm = np.asarray(left_val_430rpm)
right_val_430rpm = np.asarray(right_val_430rpm)

dt_df_430rpm = pd.DataFrame(data=dt_val_430rpm.T, columns=["red"])
left_df_430rpm = pd.DataFrame(data=left_val_430rpm.T, columns=["red"])
right_df_430rpm = pd.DataFrame(data=right_val_430rpm.T, columns=["red"])

time_430rpm = video_430rpm.get_time_line()
time_430rpm = np.asarray(time_430rpm)
time_df_430rpm = pd.DataFrame(data=time_430rpm.T, columns=["Time"])
time_df_430rpm.to_csv("all_time_430rpm.csv")
```

In [8]:
```
# Get red channel values for stirring speed 560 rpm

dt_val_560rpm, left_val_560rpm, right_val_560rpm = video_560rpm.get_red()
dt_val_560rpm = np.asarray(dt_val_560rpm)
left_val_560rpm = np.asarray(left_val_560rpm)
right_val_560rpm = np.asarray(right_val_560rpm)

dt_df_560rpm = pd.DataFrame(data=dt_val_560rpm.T, columns=["red"])
left_df_560rpm = pd.DataFrame(data=left_val_560rpm.T, columns=["red"])
right_df_560rpm = pd.DataFrame(data=right_val_560rpm.T, columns=["red"])

time_560rpm = video_560rpm.get_time_line()
time_560rpm = np.asarray(time_560rpm)
time_df_560rpm = pd.DataFrame(data=time_560rpm.T, columns=["Time"])
time_df_560rpm.to_csv("all_time_560rpm.csv")
```

In [9]:
```
# Get red channel values for stirring speed 740 rpm

video_740rpm = video_process(file_name="loop_740rpm.mp4")

dt_val_740rpm, left_val_740rpm, right_val_740rpm = video_740rpm.get_red()
dt_val_740rpm = np.asarray(dt_val_740rpm)
left_val_740rpm = np.asarray(left_val_740rpm)
right_val_740rpm = np.asarray(right_val_740rpm)

dt_df_740rpm = pd.DataFrame(data=dt_val_740rpm.T, columns=["red"])
left_df_740rpm = pd.DataFrame(data=left_val_740rpm.T, columns=["red"])
right_df_740rpm = pd.DataFrame(data=right_val_740rpm.T, columns=["red"])

time_740rpm = video_740rpm.get_time_line()
time_740rpm = np.asarray(time_740rpm)
time_df_740rpm = pd.DataFrame(data=time_740rpm.T, columns=["Time"])
time_df_740rpm.to_csv("all_time_740rpm.csv")
```

In [10]:
```
# Get red channel values for stirring speed 860 rpm

video_860rpm = video_process(file_name="loop_860rpm.mp4")

dt_val_860rpm, left_val_860rpm, right_val_860rpm = video_860rpm.get_red()
dt_val_860rpm = np.asarray(dt_val_860rpm)
left_val_860rpm = np.asarray(left_val_860rpm)
right_val_860rpm = np.asarray(right_val_860rpm)

dt_df_860rpm = pd.DataFrame(data=dt_val_860rpm.T, columns=["red"])
```

```
left_df_860rpm = pd.DataFrame(data=left_val_860rpm.T, columns=["red"])
right_df_860rpm = pd.DataFrame(data=right_val_860rpm.T, columns=["red"])

time_860rpm = video_860rpm.get_time_line()
time_860rpm = np.asarray(time_860rpm)
time_df_860rpm = pd.DataFrame(data=time_860rpm.T, columns=["Time"])
time_df_860rpm.to_csv("all_time_860rpm.csv")
```

In [11]:
```
# Set up font stype in the plots

font_style = {'fontname': 'Arial', 'size': '12'}
font_path = 'Arial.ttf'
font_prop =fm.FontProperties(fname=font_path, size=12)
```

In [12]:
```
# plot of red channel values at the right space outside of draft tube.

plt.plot(time_430rpm, right_val_430rpm, "-o", color="darkorange", label="430 rpm")
plt.plot(time_560rpm, right_val_560rpm, "-s", color="limegreen", label="560 rpm")
plt.plot(time_740rpm, right_val_740rpm, "-p", color="lightcoral", label="740 rpm")
plt.plot(time_860rpm, right_val_860rpm, "-D", color="darkviolet", label="860 rpm")
plt.xlabel("Stirring speed / rpm", **font_style)
plt.ylabel("Red channel value", **font_style)
plt.xlim(0, 12)
plt.ylim(0, 200)
plt.legend(loc=0, frameon=False, prop=font_prop)
plt.show()
```

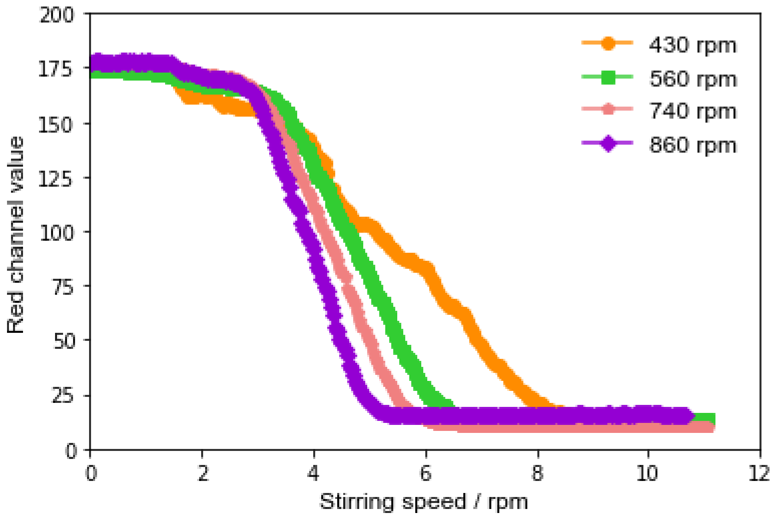

In [13]:
```
# plot of red channel values at the left space outside of draft tube.

plt.plot(time_430rpm, left_val_430rpm, "-o", color="darkorange", label="430 rpm")
plt.plot(time_560rpm, left_val_560rpm, "-s", color="limegreen", label="560 rpm")
plt.plot(time_740rpm, left_val_740rpm, "-p", color="lightcoral", label="740 rpm")
plt.plot(time_860rpm, left_val_860rpm, "-D", color="darkviolet", label="860 rpm")
plt.xlabel("Stirring speed / rpm", **font_style)
plt.ylabel("Red channel value", **font_style)
plt.xlim(0, 12)
plt.ylim(0, 200)
plt.legend(loc=0, frameon=False, prop=font_prop)
plt.show()
```

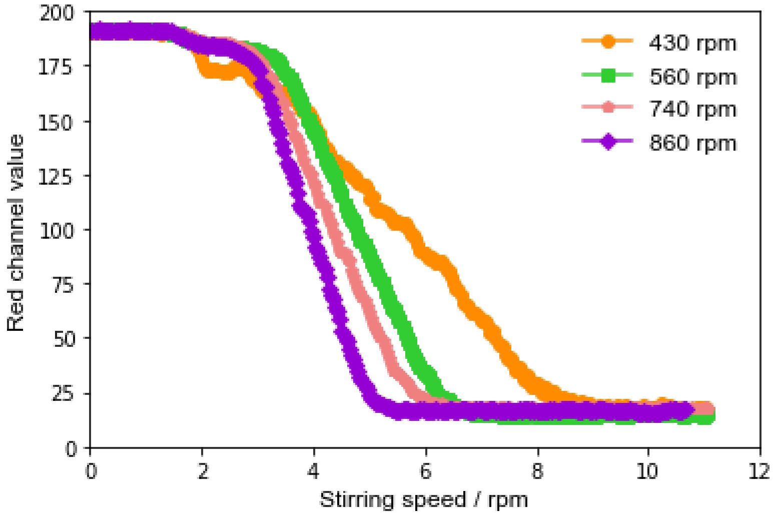


```
In [14]: # plot of red channel values in draft tube.

plt.plot(time_430rpm, dt_val_430rpm, "-o", color="darkorange", label="430 rpm")
plt.plot(time_560rpm, dt_val_560rpm, "-s", color="limegreen", label="560 rpm")
plt.plot(time_740rpm, dt_val_740rpm, "-p", color="lightcoral", label="740 rpm")
plt.plot(time_860rpm, dt_val_860rpm, "-D", color="darkviolet", label="860 rpm")
plt.xlabel("Stirring speed / rpm", **font_style)
plt.ylabel("Red channel value", **font_style)
plt.xlim(0, 12)
plt.ylim(0, 200)
plt.legend(loc=0, frameon=False, prop=font_prop)
plt.show()
```

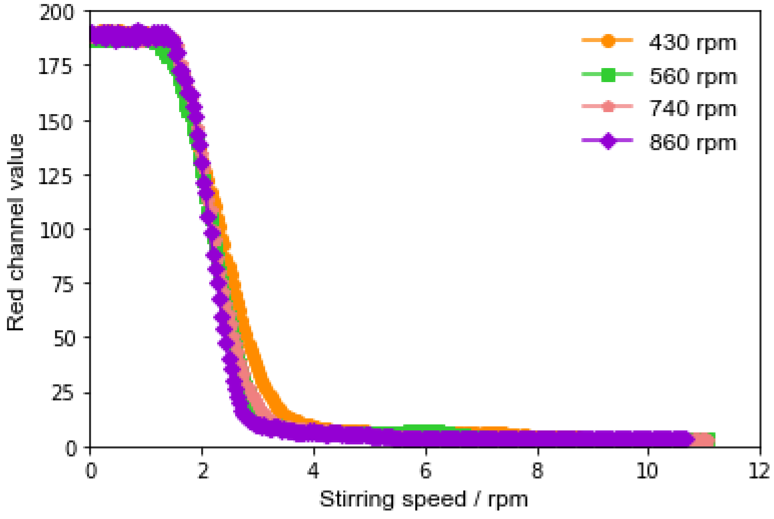

Get the plot for Figure 9 in the paper.

```
In [15]: """
430 rpm
"""
# Get the difference of red channel values to the first value in draft tube.

dt_val_430rpm_diff = dt_val_430rpm - dt_val_430rpm[0]
dt_val_430rpm_diff = np.abs(dt_val_430rpm_diff)

# Check the difference of red values, it shows when the difference starts getting bigger
# flowing into draft tube.
dt_val_430rpm_start = np.argmax(dt_val_430rpm_diff>4)
dt_430rpm_time_start = time_430rpm[dt_val_430rpm_start]

# Check the difference of red values, it shows when the difference starts getting bigger
```

```
# is complete in draft tube.
dt_val_430rpm_end = np.argmax(dt_val_430rpm_diff>181.4)
dt_430rpm_time_end = time_430rpm[dt_val_430rpm_end]

dt_time_430rpm = dt_430rpm_time_end - dt_430rpm_time_start
dt_time_430rpm = round(dt_time_430rpm,2)


# Get the difference of red channel values to the first value at right side of draft tub

right_val_430rpm_diff = right_val_430rpm - right_val_430rpm[0]
right_val_430rpm_diff = np.abs(right_val_430rpm_diff)

# the start time of fluid flow at side is the end of fluid flow in draft tube.
right_430rpm_time_start = time_430rpm[dt_val_430rpm_end]

# Check the difference of red values, it shows when the difference starts getting bigger
# is complete at outside of draft tube.
right_val_430rpm_end = np.argmax(right_val_430rpm_diff>161)
right_430rpm_time_end = time_430rpm[right_val_430rpm_end]

right_time_430rpm = right_430rpm_time_end - right_430rpm_time_start
right_time_430rpm = round(right_time_430rpm,2)
```

In [16]:

```
"""
560 rpm
"""
# Get the difference of red channel values to the first value in draft tube.

dt_val_560rpm_diff = dt_val_560rpm - dt_val_560rpm[0]
dt_val_560rpm_diff = np.abs(dt_val_560rpm_diff)

# Check the difference of red values, it shows when the difference starts getting bigger
# flowing into draft tube.
dt_val_560rpm_start = np.argmax(dt_val_560rpm_diff>4)
dt_560rpm_time_start = time_560rpm[dt_val_560rpm_start]

# Check the difference of red values, it shows when the difference starts getting bigger
# is complete in draft tube.
dt_val_560rpm_end = np.argmax(dt_val_560rpm_diff>181.9)
dt_560rpm_time_end = time_560rpm[dt_val_560rpm_end]

dt_time_560rpm = dt_560rpm_time_end - dt_560rpm_time_start
dt_time_560rpm = round(dt_time_560rpm,2)


# Get the difference of red channel values to the first value at right side of draft tub

right_val_560rpm_diff = right_val_560rpm - right_val_560rpm[0]
right_val_560rpm_diff = np.abs(right_val_560rpm_diff)

# the start time of fluid flow at side is the end of fluid flow in draft tube.
right_560rpm_time_start = time_560rpm[dt_val_560rpm_end]

# Check the difference of red values, it shows when the difference starts getting bigger
# is complete at outside of draft tube.
right_val_560rpm_end = np.argmax(right_val_560rpm_diff>159.4)
right_560rpm_time_end = time_560rpm[right_val_560rpm_end]

right_time_560rpm = right_560rpm_time_end - right_560rpm_time_start
right_time_560rpm = round(right_time_560rpm,2)
```

In [17]:

```
"""
740 rpm
"""
```

```
# Get the difference of red channel values to the first value in draft tube.

dt_val_740rpm_diff = dt_val_740rpm - dt_val_740rpm[0]
dt_val_740rpm_diff = np.abs(dt_val_740rpm_diff)

# Check the difference of red values, it shows when the difference starts getting bigger
# flowing into draft tube.
dt_val_740rpm_start = np.argmax(dt_val_740rpm_diff>4)
dt_740rpm_time_start = time_740rpm[dt_val_740rpm_start]

# Check the difference of red values, it shows when the difference starts getting bigger
# is complete in draft tube.
dt_val_740rpm_end = np.argmax(dt_val_740rpm_diff>178.6)
dt_740rpm_time_end = time_740rpm[dt_val_740rpm_end]

dt_time_740rpm = dt_740rpm_time_end - dt_740rpm_time_start
dt_time_740rpm = round(dt_time_740rpm,2)

# Get the difference of red channel values to the first value at right side of draft tub

right_val_740rpm_diff = right_val_740rpm - right_val_740rpm[0]
right_val_740rpm_diff = np.abs(right_val_740rpm_diff)

# the start time of fluid flow at side is the end of fluid flow in draft tube.
right_740rpm_time_start = time_740rpm[dt_val_740rpm_end]

# Check the difference of red values, it shows when the difference starts getting bigger
# is complete at outside of draft tube.
right_val_740rpm_end = np.argmax(right_val_740rpm_diff>166.4)
right_740rpm_time_end = time_740rpm[right_val_740rpm_end]

right_time_740rpm = right_740rpm_time_end - right_740rpm_time_start
right_time_740rpm = round(right_time_740rpm,2)
```

In [18]:

```
"""
860 rpm
"""
# Get the difference of red channel values to the first value in draft tube.

dt_val_860rpm_diff = dt_val_860rpm - dt_val_860rpm[0]
dt_val_860rpm_diff = np.abs(dt_val_860rpm_diff)


# Check the difference of red values, it shows when the difference starts getting bigger
# flowing into draft tube.
dt_val_860rpm_start = np.argmax(dt_val_860rpm_diff>4)
dt_860rpm_time_start = time_860rpm[dt_val_860rpm_start]

# Check the difference of red values, it shows when the difference starts getting bigger
# is complete in draft tube.
dt_val_860rpm_end = np.argmax(dt_val_860rpm_diff>182)
dt_860rpm_time_end = time_860rpm[dt_val_860rpm_end]

dt_time_860rpm = dt_860rpm_time_end - dt_860rpm_time_start
dt_time_860rpm = round(dt_time_860rpm,2)


# Get the difference of red channel values to the first value at right side of draft tub

right_val_860rpm_diff = right_val_860rpm - right_val_860rpm[0]
right_val_860rpm_diff = np.abs(right_val_860rpm_diff)

# the start time of fluid flow at side is the end of fluid flow in draft tube.
right_860rpm_time_start = time_860rpm[dt_val_860rpm_end]
```

```
# Check the difference of red values, it shows when the difference starts getting bigger
# is complete at outside of draft tube.
right_val_860rpm_end = np.argmax(right_val_860rpm_diff>162.4)
right_860rpm_time_end = time_860rpm[right_val_860rpm_end]

right_time_860rpm = right_860rpm_time_end - right_860rpm_time_start
right_time_860rpm = round(right_time_860rpm,2)
```

In [19]:

```
# Here are the quantified mixing time inside and outside of draft tube with different mi

stirring_speed = np.asarray([430, 560, 740, 860])
dt_time = np.asarray([dt_time_430rpm, dt_time_560rpm, dt_time_740rpm, dt_time_860rpm])
side_time = np.asarray([right_time_430rpm, right_time_560rpm, right_time_740rpm, right_t

# Fit the values with an exponential function.

def exponential(x, a, b):
    return a * np.power(x, b)

pars_dt, cov_dt = curve_fit(f=exponential, xdata=stirring_speed, ydata=dt_time, p0=[0,0]
pars_side, cov_side = curve_fit(f=exponential, xdata=stirring_speed, ydata=side_time, p0

sp = np.arange(425, 865)

fit_dt_val = exponential(sp, *pars_dt)
fit_side_val = exponential(sp, *pars_side)

x_ticks = [430, 560, 740, 860]
bar_width = 20

plt.bar(stirring_speed - bar_width/2, dt_time, width=bar_width, color="r", label="draft
plt.bar(stirring_speed + bar_width/2, side_time, width=bar_width, color="b", label="side
#plt.plot(sp, fit_dt_val, "--r", label="fitted draft tube")
#plt.plot(sp, fit_side_val, "--b", label="fitted side")
plt.xticks(x_ticks)
plt.xlabel("Stirring speed / rpm", **font_style)
plt.ylabel("Time / s", **font_style)
plt.legend(loc=0, frameon=False, prop=font_prop)
plt.show()
```

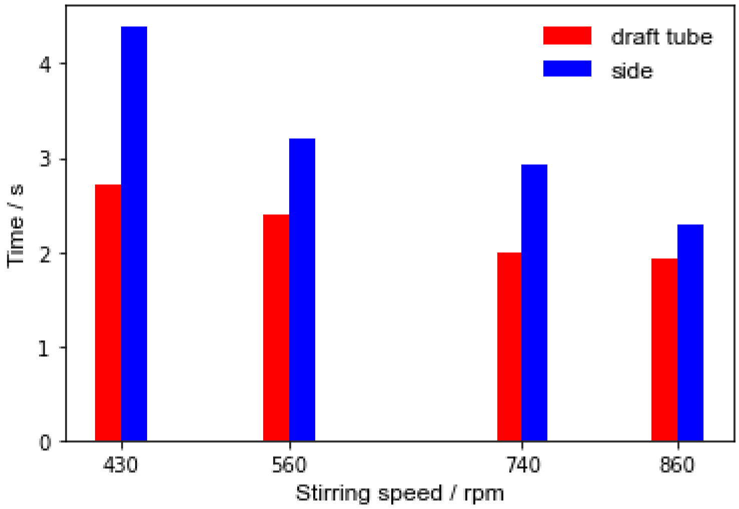

Supplement: File 3 — Python code for video processing to get mixing time. [file Beilstein_J_Org_Chem-20-74-s003.zip › SI3_video_processing.pdf]
